# GDF15 induces excessive activation of osteoclasts within the vertebral endplates leading to early endplate degeneration

**DOI:** 10.1172/jci.insight.190598

**Published:** 2025-11-11

**Authors:** Xiaoqun Li, Jinhui Wu, Qingjie Kong, Miao Hu, Yuhong Li, Ziheng Wei, Heng Jiang, Xuhui Zhou, Jun Ma

**Affiliations:** 1 Department of Orthopedics, Shanghai Changhai Hospital, Naval Medical University, Shanghai, China.; 2 Department of Orthopedics, Shanghai Changzheng Hospital, Naval Medical University, Shanghai, China.; 3 Department of Orthopedics, Shanghai General Hospital, Shanghai Jiao Tong University School of Medicine, Shanghai, China.

**Keywords:** Aging, Bone biology, Mouse models, Osteoclast/osteoblast biology

## Abstract

Modic type 1 and 2 changes (MC-1 and MC-2) are highly prevalent in individuals with chronic low back pain, yet the cellular and molecular mechanisms underlying vertebral endplate degeneration remain poorly defined. Here, we report that osteoclastogenesis is markedly elevated in MC-1 and MC-2 lesions compared with MC-3 lesions, suggesting an active role for osteoclasts in the early stages of degeneration. Using a lumbar spine instability (LSI) mouse model, we demonstrate enhanced osteoclast activity in degenerating endplates. RNA sequencing of mononuclear cells isolated from the endplate and adjacent subchondral bone identified *Gdf15* as a potential upstream regulator of this process. Conditional knockout of *Gdf15* in monocytes reduced osteoclast formation, aberrant CD31^hi^Emcn^hi^ angiogenesis, and pain-associated neurogenesis, ultimately mitigating endplate degeneration and mechanical allodynia. Mechanistically, GDF15 promoted the fusion of preosteoclasts by modulating the expression of Rho family small GTPases. In a humanized GDF15 knockin mouse model, therapeutic neutralization of GDF15 led to a reduction in osteoclast burden, improved endplate structure, and attenuated pain behavior. Together, these findings uncover a previously unrecognized role for GDF15 in driving osteoclast-mediated endplate degeneration and highlight its potential as a therapeutic target for the treatment of endplate-related chronic low back pain.

## Abstract 2

GDF15 induces excessive activation of osteoclasts within the vertebral endplates leading to early endplate degeneration

## Introduction

Chronic low back pain (CLBP) is a significant social health and clinical issue, affecting over 80% of people worldwide at some point in their lives, particularly the elderly (1–3). CLBP is one of the leading causes of total global years lived with disability (4). However, the pathogenesis of this multifactorial condition remains unknown. Current clinical therapeutic options for CLBP primarily include conservative treatments and surgical interventions, but neither are fully satisfactory. Therefore, it is essential to explore the pathogenesis and underlying mechanisms of CLBP to develop more effective therapies.

Clinically, CLBP resulting from endplate injuries is quite common. Epidemiological studies have shown that the incidence rate of endplate injuries in individuals with CLBP is approximately 30% (5). Modic changes (MCs), also known as Modic type endplate changes, are MRI-detectable alterations in the vertebral body adjacent to the endplates of the intervertebral discs. These changes are classified into three types based on their appearance on MRI. Modic type 1 changes (MC-1) are characterized by a hypointense signal on T1-weighted images and a hyperintense signal on T2-weighted images, indicating bone marrow edema and inflammation. Modic type 2 changes (MC-2) show a hyperintense signal on both T1- and T2-weighted images, suggesting fatty degeneration of the bone marrow. In certain cases, such as trauma, infection, or accelerated degeneration, MC-2 may transform into MC-1 changes, accompanied by overlapping alterations. Finally, Modic type 3 changes (MC-3) are identified by a hypointense signal on both T1- and T2-weighted images, indicating sclerosis of the bone marrow. MC1 and MC2 types can transform into each other over time, ultimately progressing to MC3. However, the rate of reverse transformation is relatively low, and several studies have demonstrated that the early stages of endplate degeneration (MC-1 and MC-2), particularly MC-1, are more prevalent in patients with CLBP compared with asymptomatic individuals (6, 7). This suggests a correlation between early endplate degeneration and the presence of back pain.

The osteogenesis-related capillary subtype known as type H vessels, which is characterized by high expression of CD31 and endomucin (CD31^hi^Emcn^hi^), was proved to couple with osteogenesis and angiogenesis (8). An increased presence of type H vessels in the endplates, potentially associated with osteoclast activity, has been observed in degenerative conditions and may contribute to endplate sclerosis and low back pain (LBP) (9). The innervation of the vertebral body and endplate primarily originates from the basivertebral nerve, a branch of the sinuvertebral nerve (10). The basivertebral nerve exhibits positive reactions for substance P and PGP 9.5, indicating its capability to transmit pain (11, 12). Under physiological conditions, the nerve density in the endplate is similar to that in the outer annulus fibrosus; however, under pathological conditions, the nerve density in the endplate is higher than that in nonpathological endplates and the pathological annulus fibrosus (13, 14). Recent studies have shown that osteoclast senescence contributes to sensory innervation and pain in degenerating endplates (9). However, the upstream signals responsible for initiating osteoclast activation during the early stages of endplate degeneration remain incompletely understood.

Growth differentiation factor 15 (GDF15) is a member of the TGF-β family that has been demonstrated to play an important role in several pathological states, such as inflammatory bone loss, arthritis, and rheumatoid arthritis etc. (15–18). Under normal conditions, GDF15 is expressed at low levels in most tissues, but its expression increases in pathological conditions such as tissue injury or inflammation (19–21). It has been reported that GDF15 can regulate bone metabolic balance through multiple pathways. Siddiqui et al. reported that GDF15 enhances osteoblast function and promotes the growth of prostate cancer in bone by activating osteoclastogenesis through the osteoblastic production of CCL2 and RANKL and the recruitment of osteomas. Additionally, a study showed that hypoxia can induce the expression of GDF15 and activate the NF-κB pathway, which promotes osteoclast differentiation (22). Furthermore, GDF15 has been found to enhance osteoclastogenesis in human peripheral blood monocytes and facilitate tumor progression (23, 24). However, the specific mechanisms by which GDF15 affects osteoclast differentiation in endplates during the early stages of MCs remain largely unexplored.

Therefore, in this study, we report the overactivation of osteoclastogenesis in the early stages of MCs. We identified that *Gdf15* is overexpressed in monocytes within the endplates during early endplate degeneration, which initiates the fusion of osteoclasts via regulating the expression of small GTPases of Rho family. GDF15 knockout in the monocytes attenuated the overactivation of osteoclasts and endplate degeneration. Furthermore, we demonstrated that the neutralization of GDF15 could reduce the overactivation of osteoclasts, type H blood vessel formation, and sensory nerve ingrowth in endplates, thereby alleviating symptoms in the early stages of MCs. In summary, our study suggests that GDF15 contributes to the progression of early endplate degeneration by activating osteoclasts in the endplates. Targeting GDF15 represents a potential therapeutic approach for treating MC-related LBP.

## Results

### Bone resorption activity was found to be more pronounced in patients with MC-1 and MC-2 changes.

**Figure 1:**
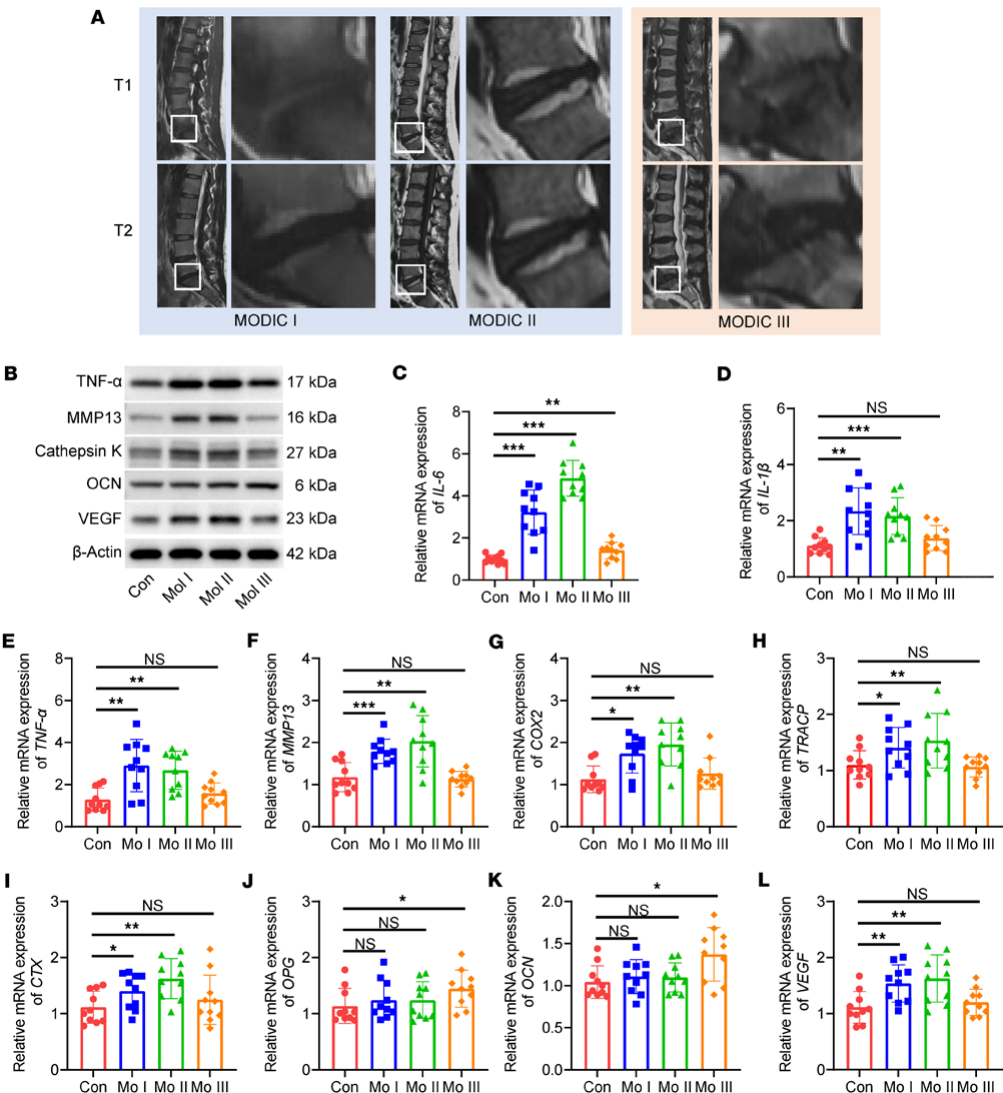
Upregulated expression of bone resorption–associated genes in patients with Modic type 1 and type 2 changes. (**A**) Representative sagittal T1- and T2-weighted MRI images from patients exhibiting Modic type 1, type 2, and type 3 changes. (**B**) Western blot analysis of protein expression of TNF-α, MMP13, cathepsin K, OCN, and VEGF in endplate tissues. (**C**–**L**) Quantitative RT-PCR analysis of mRNA expression levels of proinflammatory cytokines *IL-6* (**C**), *IL-1β* (**D**), *TNF-α* (**E**); cartilage catabolic markers *MMP13* (**F**) and *COX2* (**G**); bone resorption markers *TRACP* (**H**) and *CTX* (**I**); bone formation markers *OPG* (**J**) and *OCN* (**K**); and angiogenic factor *VEGF* (**L**) in endplate tissues obtained from patients with different Modic types and individuals in the control group. Data are presented as mean ± SD. Statistical analysis was performed using 2-tailed ANOVA with Tukey’s test for differences among groups. *P < 0.05, ^**^P < 0.01, ***P < 0.001.

MCs, identifiable on MRI, represent pathological alterations in the vertebral bone marrow adjacent to the cartilaginous endplates of intervertebral discs. Modic type 1 is characterized by hypointense signals on T1-weighted images and hyperintense signals on T2-weighted images, indicative of bone marrow edema and inflammation. In contrast, Modic type 2 exhibits hyperintense signals on both T1- and T2-weighted images, reflecting fatty replacement of the bone marrow. Modic type 3 is marked by hypointense signals on both T1- and T2-weighted images, corresponding to bone sclerosis. In this study, endplate tissues were harvested from patients with CLBP undergoing lumbar spinal fusion surgery. Based on preoperative MRI findings, samples were stratified into 4 groups: control (no MCs), Modic type 1, Modic type 2, and Modic type 3 (Figure 1A).

Gene expression analyses were subsequently performed on the collected endplate tissues. The expression levels of proinflammatory cytokines — *IL-6*, *IL-1β*, and *TNF-* — were elevated in the Modic type 1 group and progressively decreased in Modic types 2 and 3, aligning with the degree of inflammatory activity observed in imaging (Figure 1, C–E). Markers of cartilage degradation, *MMP13* and *COX2*, were most highly expressed in Modic type 2 samples, suggesting peak cartilage matrix destruction at this stage (Figure 1, F and G). Analysis of bone metabolism markers revealed that bone resorption indicators — C-terminal telopeptide of type I collagen (*CTX*) and tartrate-resistant acid phosphatase (*TRACP*) — were upregulated in Modic types 1 and 2, while they declined in Modic type 3. Conversely, markers of bone formation — osteocalcin (OCN) and osteoprotegerin (*OPG*) — showed only a modest increase in Modic type 3, implying a transition from bone resorption to sclerosis during the course of degeneration (Figure 1, H–K). Additionally, *VEGF*, a key mediator of angiogenesis, demonstrated the highest expression in Modic type 1 and the lowest in Modic type 3, indicating elevated bone remodeling activity in the early stages of endplate degeneration (Figure 1L).

To further validate these findings, we performed Western blot analysis to assess the protein expression levels of TNF-α, MMP13, cathepsin K, OCN, and VEGF. The results corroborated the transcriptional data, highlighting increased inflammatory and degenerative activity, along with enhanced osteoclastogenesis and angiogenesis during the early phases of endplate degeneration. In contrast, late-stage degeneration was associated with a modest upregulation of osteogenic markers, suggesting a shift toward bone formation and sclerosis (Figure 1B).

### Lumbar spine instability induces early endplate degeneration in a murine model.

Previous studies have demonstrated that mechanical instability of the spinal facet joints can lead to aberrant osteoclast activation and subsequent degeneration of the vertebral endplates (9, 25). To elucidate the pathological mechanisms involved in early-stage endplate degeneration, we established a lumbar spine instability (LSI) mouse model by performing bilateral facet joint transection. This model mimics mechanical instability–induced stress on the vertebral column and facilitates the investigation of degenerative changes in the intervertebral unit.

**Figure 2:**
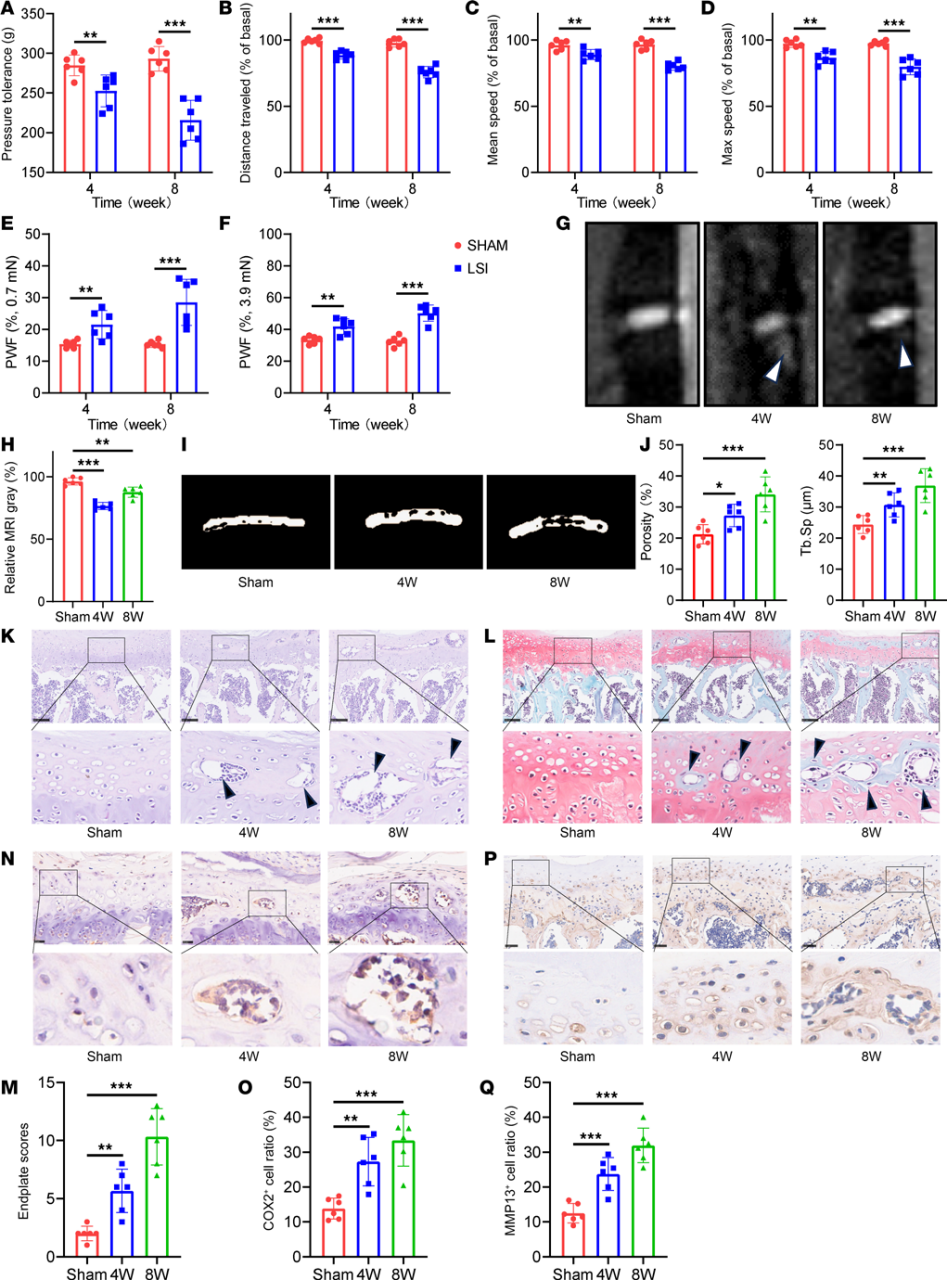
Lumbar spine instability induces early endplate degeneration in mice. (**A**–**D**) Evaluation of spontaneous locomotor activity at various time points following LSI surgery using behavioral metrics: pressure tolerance (**A**), total distance traveled (**B**), mean velocity (**C**), and maximum speed (**D**). (**E** and **F**) Mechanical allodynia was assessed by measuring hind paw withdrawal frequency in response to von Frey filament stimulation (0.7 mN and 3.9 mN) following sham or LSI surgery. (**G**) Representative sagittal T2-weighted MRI images of lumbar spine from sham and LSI mice at indicated time point. Arrows indicate the high signal. (**H**) Quantitative MRI signal intensity analysis of endplate regions. (**I**) Representative 3D micro-CT reconstructions of lumbar vertebral endplates in sham and LSI mice. (**J**) Quantitative micro-CT analysis of endplate total porosity and trabecular separation (Tb.Sp). (**K** and **L**) Representative histological images of endplates stained with H&E (**K**) and safranin O/fast green (**L**). Arrows indicate the degeneration area. Scale bars: 100 μm. (**M**) Quantification of endplate degeneration scores based on safranin O/fast green staining. (**N** and **P**) Immunohistochemical staining of COX2 (**N**) and MMP13 (**P**) in endplates from sham and LSI mice. Scale bars: 50 μm. (**O** and **Q**) Quantification of COX2^+^ (**O**) and MMP13^+^ (**Q**) cells per field of view. Data are presented as mean ± SD. Statistical analysis was performed using 2-tailed Student’s *t* tests (**A**–**F**) or 2-tailed ANOVA with Tukey’s test (**H**, **J**, **M**, **O**, and **Q**) for differences among groups. *P < 0.05, **P < 0.01, ***P < 0.001.

Behavioral assessments were conducted to evaluate mechanical hyperalgesia associated with LBP following LSI surgery. Mice subjected to LSI exhibited significant pain-like behaviors, with maximal responses observed at 8 weeks after operation, indicating the development of chronic lumbar hyperalgesia (Figure 2, A–F). T2-weighted MRI revealed the emergence of high signal intensity regions beneath the endplates at 4 and 8 weeks in LSI-treated mice, suggestive of inflammatory changes characteristic of early endplate degeneration (Figure 2, G and H). Micro-computed tomography (micro-CT) analysis further confirmed progressive deterioration of endplate architecture in the LSI group, evidenced by increased endplate porosity and a marked rise in trabecular separation (Tb.Sp) over time (Figure 2, I and J). Histological evaluation using H&E as well as safranin O/fast green staining demonstrated notable structural disruption, including endplate perforation and sclerosis, at both 4 and 8 weeks following LSI surgery (Figure 2, K–M).

Immunohistochemical and PCR analysis revealed upregulated expression of cartilage catabolic enzymes MMP13 and COX2 in the endplates of LSI mice, with peak levels detected at 8 weeks. Quantitative analysis confirmed the temporal pattern of inflammatory and catabolic activity, reinforcing the role of inflammation-mediated cartilage degradation during the early phase of degeneration (Figure 2, N–Q and Supplemental Figure 1, A and B; supplemental material available online with this article; https://doi.org/10.1172/jci.insight.190598DS1). Collectively, these findings provide compelling evidence that LSI induces progressive structural and molecular changes in vertebral endplates, culminating in early degenerative alterations within 8 weeks after surgery.

### Osteoclast overactivation and GDF15 upregulation coordinate early endplate degeneration.

**Figure 3:**
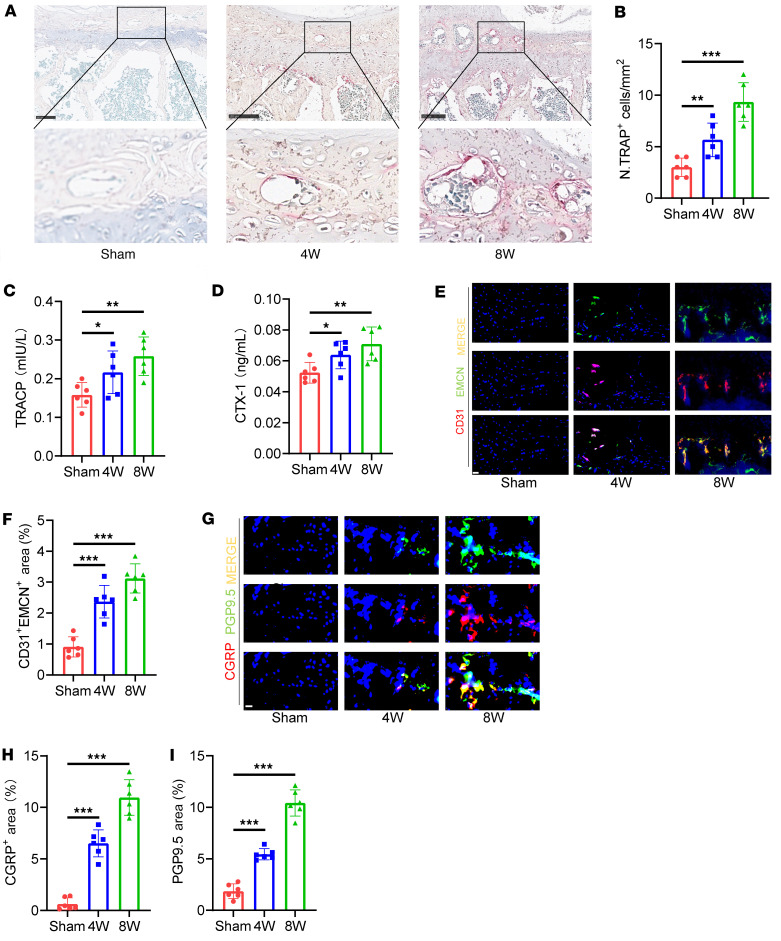
Osteoclastogenesis is overactivated in the endplates of LSI mice. (**A**) Representative images of TRAP staining in lumbar endplates from LSI and sham mice. Scale bars: 100 μm. (**B**) Quantification of TRAP^+^ osteoclasts per endplate section. (**C** and **D**) Serum levels of osteoclast activity markers, TRACP (**C**) and CTX-1 (**D**), measured at the indicated time points after surgery. (**E**) Representative immunofluorescence images showing CD31 (red), endomucin (Emcn, green), and merged CD31^hi^Emcn^hi^ (yellow) type H vessels in endplates of LSI and sham mice. Scale bars: 20 μm. (**F**) Quantitative analysis of CD31^hi^Emcn^hi^ vessel area in the endplates. (**G**) Representative immunofluorescence staining of sensory nerve markers PGP9.5 and CGRP in endplates of LSI and sham mice. Scale bars: 10 μm. (**H** and **I**) Quantitative analysis of PGP9.5^+^ (**H**) and CGRP^+^ (**I**) fiber area in endplates. Data are presented as mean ± SD. Statistical analysis was performed using 2-tailed ANOVA with Tukey’s test for differences among groups. *P < 0.05, **P < 0.01, ***P < 0.001.

To investigate the role of osteoclast-mediated bone resorption in the early stages of endplate degeneration, we first assessed osteoclast distribution via tartrate-resistant acid phosphatase (TRAP) staining. The results demonstrated a marked increase in the number of TRAP^+^ osteoclasts in the endplates following LSI surgery, with the highest number observed at 8 weeks after surgery (Figure 3, A and B). To further evaluate osteoclastogenesis, we quantified key bone resorption markers — TRACP and C-terminal telopeptide of type I collagen (CTX-1) — using ELISA and RT-PCR. Both markers were elevated at 4 and 8 weeks after LSI, confirming enhanced osteoclast differentiation and activity during this period (Figure 3, C and D, and Supplemental Figure 2, A and B). We next examined the presence of type H vessels — specialized capillaries characterized by high expression of platelet endothelial cell adhesion molecule-1 (CD31) and endomucin (EMCN) — which are known to be associated with osteoclast activity and couple angiogenesis with bone remodeling. Immunofluorescence staining revealed an increase in CD31^hi^Emcn^hi^ vessels in the endplates of LSI-treated mice compared with sham-operated controls (Figure 3, E and F). Given that osteoclasts have been implicated in nociceptive signaling through the promotion of sensory nerve growth, we also assessed pain-related nerve fiber innervation. Immunofluorescence staining showed increased expression of the pan-neuronal marker PGP9.5 and the nociceptive neuropeptide CGRP in the endplates of LSI mice, particularly at 8 weeks, indicating enhanced sensory innervation associated with pain generation during early degeneration (Figure 3, G–I). Collectively, these findings suggest that early endplate degeneration is characterized by pathological overactivation of osteoclasts, accompanied by vascular and sensory remodeling.

To further validate the role of osteoclast overactivation in early endplate degeneration, we administered alendronate, a pharmacological inhibitor of osteoclast activity, to suppress osteoclast overactivation within the vertebral endplates following LSI surgery (Supplemental Figure 3, A–D). Compared with the untreated LSI group, mice treated with alendronate exhibited a reduction in the number of type H vessels in the endplates at both 4 and 8 weeks after surgery (Supplemental Figure 3, E and F). Histological assessments using safranin O/fast green and H&E staining demonstrated that inhibition of osteoclast activity markedly preserved endplate structural integrity, with improved histological scores (Supplemental Figure 3, G–I). Furthermore, RT-PCR analysis revealed that expression levels of cartilage catabolic markers *Cox2* and *Mmp13* were substantially reduced following osteoclast inhibition, indicating attenuation of cartilage matrix degradation (Supplemental Figure 3, J and K). Consistently, T2-weighted MRI scans showed a notable reduction in abnormally elevated signal intensity beneath the endplates, suggesting alleviation of inflammation-associated degeneration (Supplemental Figure 3, L and M). Micro-CT analysis further supported these findings, showing decreased endplate porosity and a reduction in Tb.Sp over time in the alendronate-treated group (Supplemental Figure 3, N–O). Collectively, these results strongly indicate that excessive osteoclast activation is a key pathological driver of early endplate degeneration, and its inhibition can effectively mitigate structural and molecular deterioration of the endplate.

**Figure 4:**
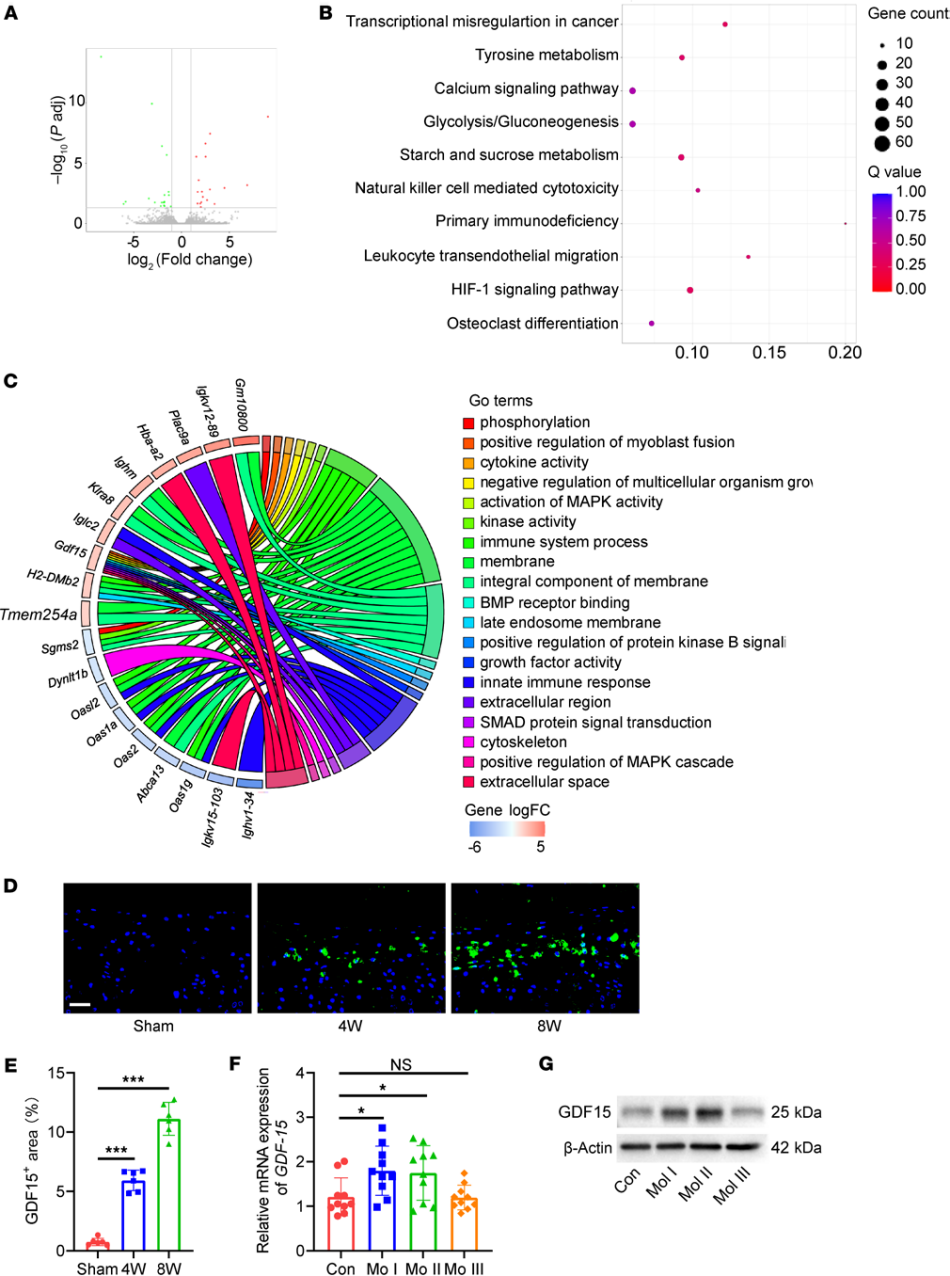
GDF15 is upregulated in the endplates of LSI mice. (**A**) Volcano plot of differentially expressed genes (DEGs) in monocytes isolated from endplates of LSI vs. sham mice. (**B**) Gene Ontology (GO) enrichment analysis of upregulated DEGs. (**C**) GOChord plot linking selected GO terms with corresponding DEGs. (**D**) Representative immunofluorescence images of GDF15^+^ cells in endplates. Scale bars: 20 μm. (**E**) Quantitative analysis of GDF15^+^ cell area in endplates. (**F**) Relative mRNA expression of *Gdf15* in endplates from LSI and sham mice. (**G**) Western blot analysis of GDF15 protein levels in human endplate tissues across different Modic types. Data are presented as mean ± SD. Statistical analysis was performed using 2-tailed ANOVA with Tukey’s test for differences among groups. *P < 0.05, ***P < 0.001.

To further delineate the molecular changes underlying osteoclast overactivation, we performed RNA sequencing of monocytes isolated from the endplate and subchondral bone of both LSI-treated and sham-operated mice. Differential gene expression analysis revealed 272 upregulated and 523 downregulated genes in the LSI group compared with the control group (Figure 4A). Gene Ontology (GO) enrichment analysis indicated that the upregulated genes were primarily involved in NF-κB signaling, MAPK pathways, and bone resorption processes (Figure 4B). Kyoto Encyclopedia of Genes and Genomes (KEGG) pathway analysis confirmed marked enrichment of genes related to osteoclast differentiation in the LSI group (Supplemental Figure 4A). Among the differentially expressed genes, *Gdf15* emerged as the most markedly upregulated gene associated with osteoclastogenesis (log_2_ fold change > 1.5; Supplemental Figure 4B). GOChord analysis revealed that *Gdf15* participates in over 40 biological processes and is positively correlated with osteoclast-related pathways (Figure 4C). Temporal expression analysis showed that GDF15 levels were elevated at 4 weeks after LSI and further increased at 8 weeks (Figure 4, D and E). To assess the clinical relevance of these findings, we examined GDF15 expression in endplate tissues from patients with MCs. RT-PCR and Western blot analysis showed that GDF15 expression was markedly increased in endplates from patients with MC-1 and MC-2 changes, further supporting its involvement in early-stage degeneration (Figure 4, F and G).

In summary, these results demonstrate that early endplate degeneration is characterized by excessive osteoclast activation and upregulation of GDF15, which may contribute to both bone resorption and associated inflammatory and nociceptive changes.

### Conditional knockout of Gdf15 inhibited osteoclastogenesis in endplates and alleviated the symptoms of early endplate degeneration in mice.

To investigate the functional role of *Gdf15* in osteoclastogenesis and early endplate degeneration, we generated monocyte-specific *Gdf15* conditional knockout mice (Lysm^cre^; *Gdf15^fl/fl^*; *Gdf15* cKO mice). Immunofluorescence staining confirmed successful deletion of *Gdf15* in monocyte-lineage cells within the endplate tissue (Supplemental Figure 5A). Importantly, *Gdf15* cKO mice exhibited comparable body weight, body length, and organ development relative to *Gdf15^fl/fl^* controls, indicating that monocyte-specific deletion of *Gdf15* does not impair general development or systemic health (Supplemental Figure 5, B–D).

**Figure 5:**
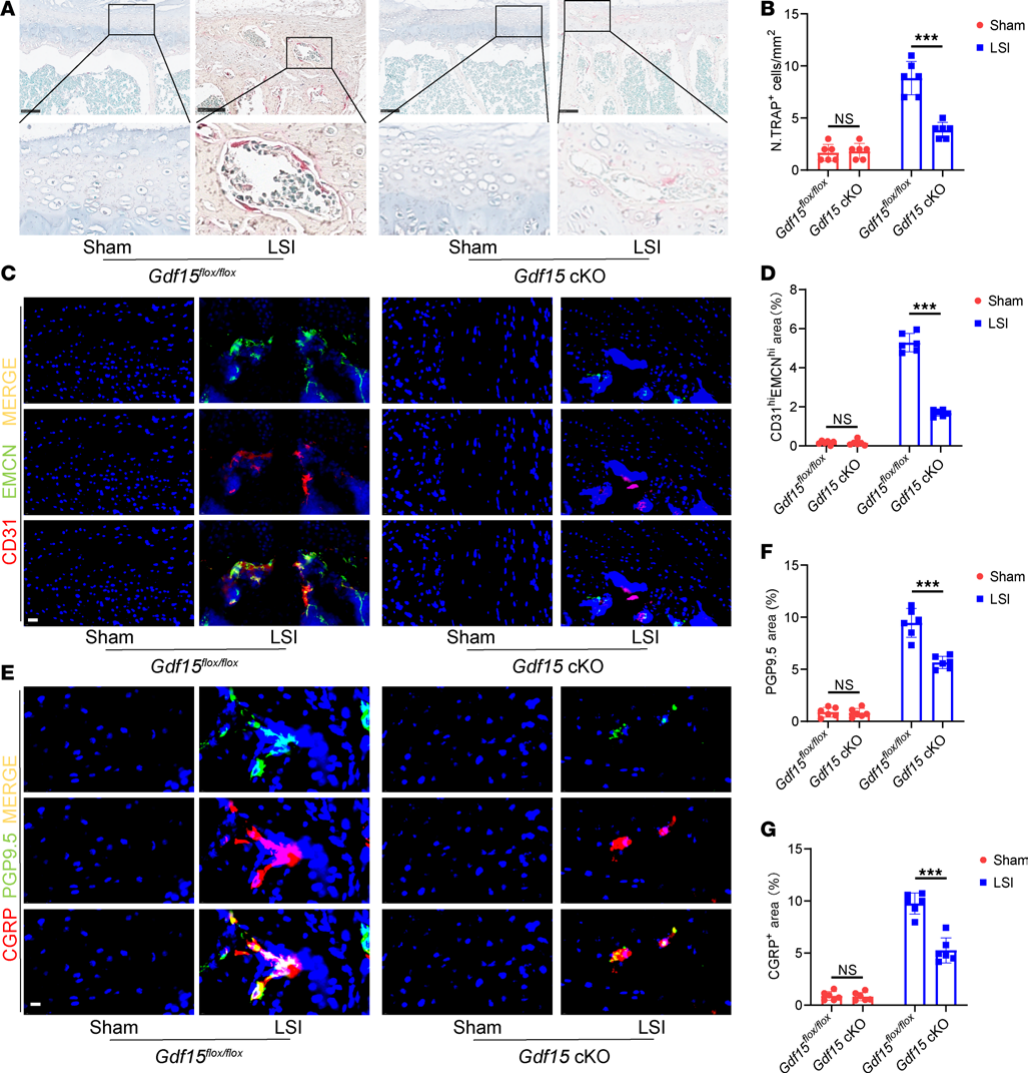
Conditional knockout of *Gdf15* inhibits osteoclastogenesis in the endplates of mice. (**A**) Representative images of TRAP staining showing osteoclast activity in endplates of *Gdf15*^fl/fl^ and *Gdf15* cKO (LysM-Cre; *Gdf15*^fl/fl^) mice following LSI or sham surgery. Scale bars: 100 μm. (**B**) Quantification of TRAP^+^ osteoclasts in the endplates. (**C**) Representative immunofluorescence staining for CD31 (red), endomucin (green), and merged CD31^hi^Emcn^hi^ (yellow) type H vessels in endplates. Scale bars: 20 μm. (**D**) Quantitative analysis of the area occupied by CD31^hi^Emcn^hi^ vessels in the endplate. (**E**) Representative immunofluorescence images of sensory nerve markers CGRP and PGP9.5 in *Gdf15^fl/fl^* and *Gdf15* cKO mice. Scale bars: 10 μm. (**F** and **G**) Quantitative analysis of CGRP^+^ (**F**) and PGP9.5^+^ (**G**) nerve fibers in the endplate region. Data are presented as mean ± SD. Statistical analysis was performed using 2-tailed Student’s *t* tests. ***P < 0.001.

Behavioral analysis revealed that *Gdf15* cKO alleviated mechanical hypersensitivity and pain-like behaviors associated with early endplate degeneration following LSI surgery (Supplemental Figure 6). Histological TRAP staining showed a marked reduction in the number of TRAP^+^ osteoclasts within the endplates of *Gdf15* cKO mice after LSI surgery (Figure 5, A and B). Additionally, the osteoclast activity markers, TRACP-5b and CTX-1, were lower in the *Gdf15* cKO group (Supplemental Figure 7, A–D).

Immunohistochemical staining revealed a decrease in the number of type H vessels within the endplates of *Gdf15* cKO mice compared with *Gdf15^fl/fl^* controls after LSI, suggesting reduced angiogenesis associated with osteoclast activity (Figure 5, C and D). Furthermore, expression of sensory nerve markers PGP9.5 and CGRP was also reduced in the endplates of Gdf15-deficient mice, indicating impaired sensory innervation during degeneration (Figure 5, E–G).

**Figure 6:**
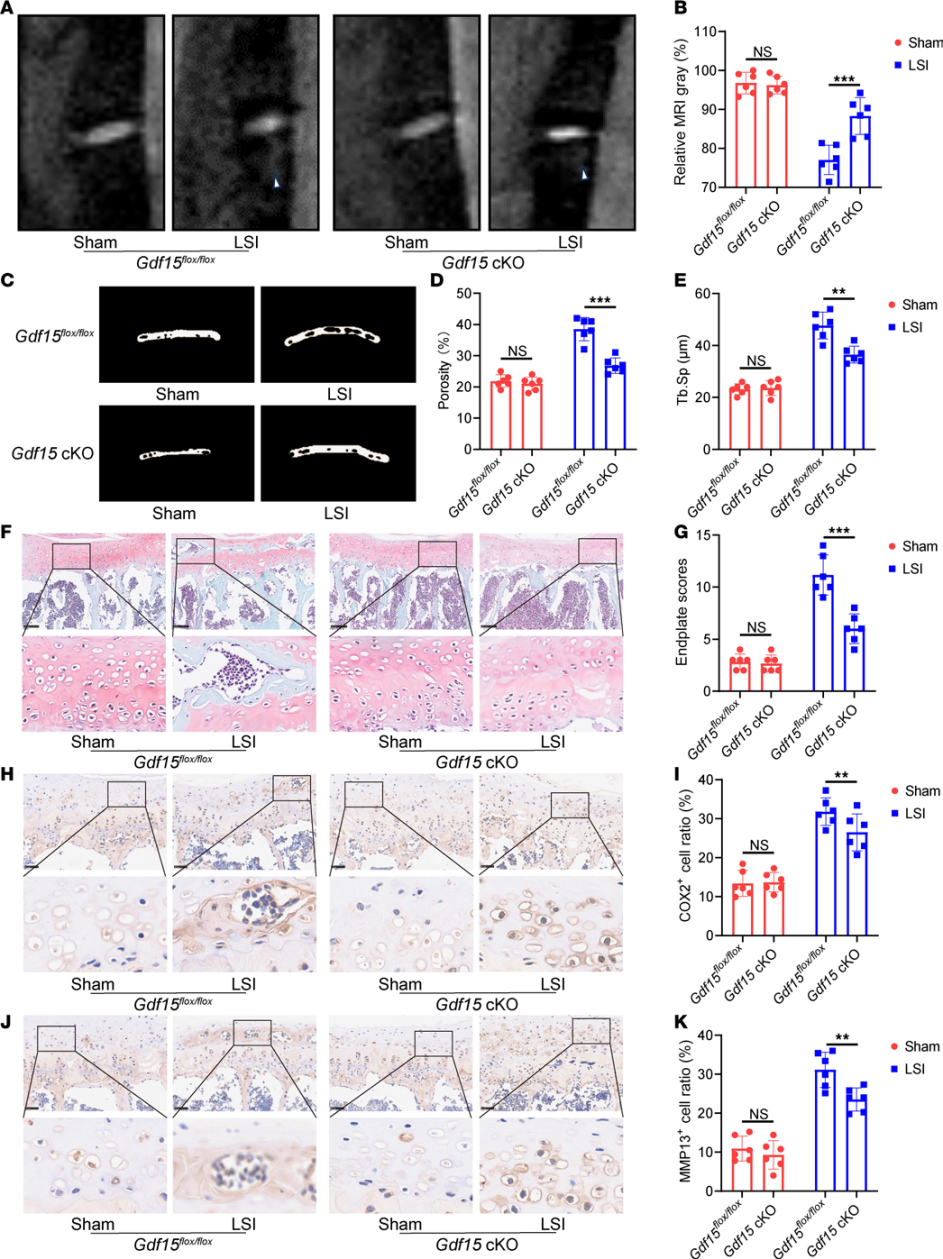
Conditional knockout of *Gdf15* attenuates endplate degeneration in mice. (**A**) Representative T2-weighted MRI images of lumbar spines in *Gdf15^fl/fl^* and *Gdf15* cKO mice after LSI surgery. Arrows indicate the high signal. (**B**) Quantitative analysis of MRI signal intensity reflecting inflammatory changes in the endplates. (**C**) Representative 3D micro-CT reconstructions of lumbar endplates. (**D** and **E**) Quantitative micro-CT analysis of endplate total porosity and trabecular separation (Tb.Sp). (**F**) Representative histological images of endplates stained with safranin O/fast green. Scale bars: 100 μm. (**G**) Histological scoring of endplate degeneration based on safranin O/fast green staining. (**H**) Representative images of COX2 staining. Scale bars: 50 μm. (**I**) Quantitative analysis of COX2^+^ cells. (**J**) Representative images of MMP13 staining after treatment with control IgG or anti-GDF15 mAb of sham mice and mice after LSI surgery. Scale bars: 100 μm. (**K**) Quantitative analysis of MMP13^+^ cells. Data are presented as mean ± SD. Statistical analysis was performed using 2-tailed Student’s *t* tests. **P < 0.01, ***P < 0.001.

To further assess the structural effects of *Gdf15* deletion, we performed T2-weighted MRI and micro-CT analyses. MRI scans showed that *Gdf15* knockout alleviated the abnormal hyperintense signal beneath the endplates, indicative of reduced inflammation (Figure 6, A and B). Micro-CT analysis demonstrated reductions in endplate porosity and Tb.Sp in *Gdf15* cKO mice compared with WT counterparts (Figure 6, C–E).

Histological analysis using H&E and safranin O/fast green staining further confirmed that *Gdf15* deletion preserved endplate structural integrity and reduced degeneration scores (Figure 6, F and G, and Supplemental Figure 8). In addition, immunohistochemistry and quantitative PCR revealed decreased expression of cartilage catabolic markers MMP13 and COX2 in the *Gdf15* cKO group, suggesting attenuation of matrix degradation (Figure 6, H–K, and Supplemental Figure 9, A and B).

Taken together, these findings demonstrate that monocyte-specific deletion of *Gdf15* suppresses osteoclastogenesis and associated pathological features — including angiogenesis, sensory nerve ingrowth, and matrix degradation — thereby mitigating early endplate degeneration in mice.

### Gdf15 promotes the fusion of preosteoclasts by regulating the expression of small Rho GTPases.

**Figure 7:**
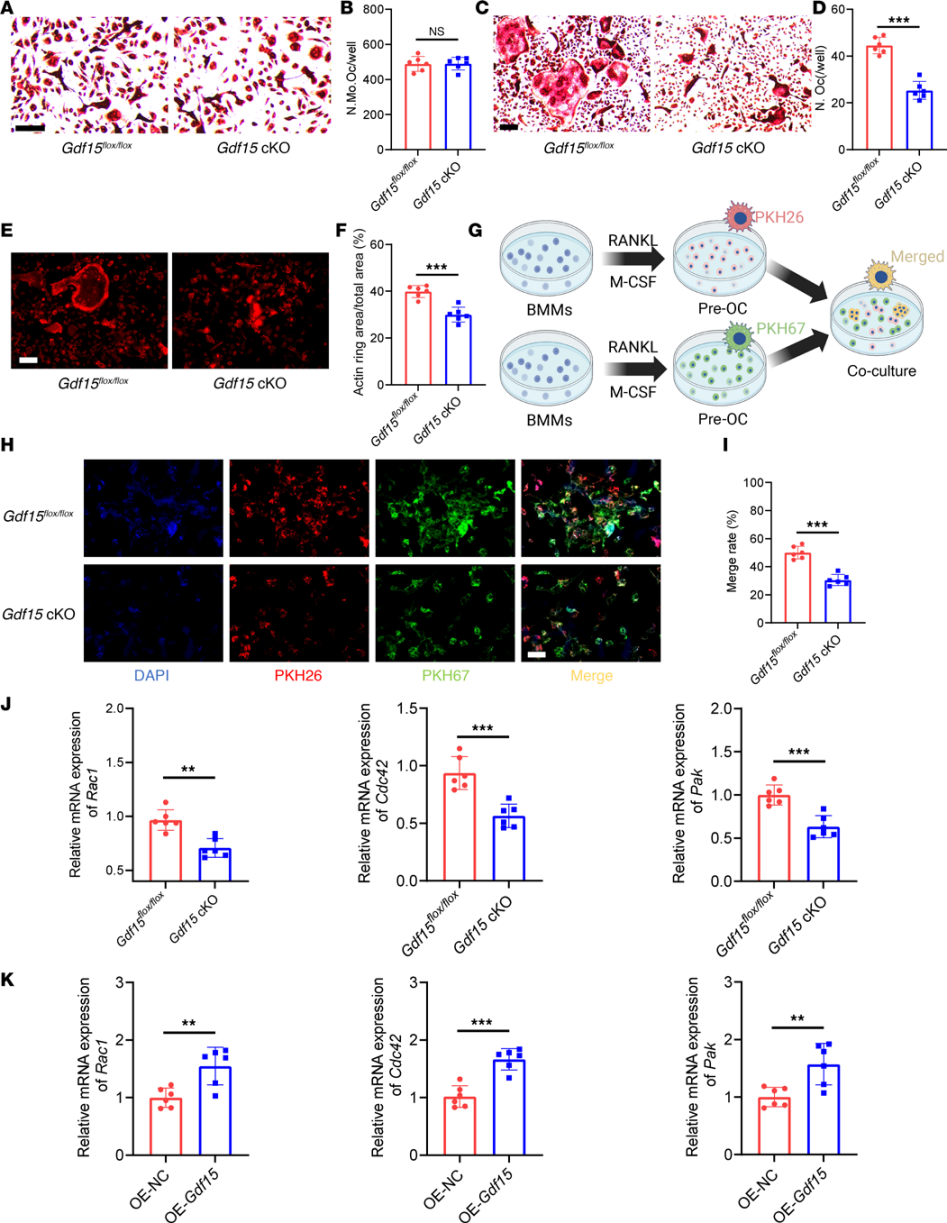
GDF15 regulates preosteoclast fusion by modulating the expression of small rho GTPases. (**A**) Representative TRAP staining images of bone marrow–derived mononuclear cells (BMMs) from *Gdf15^fl/fl^* and *Gdf15* cKO mice after 3 days of RANKL induction, highlighting mononuclear TRAP^+^ osteoclast precursors. Scale bar: 50 μm. (**B**) Quantitative analysis of mononuclear TRAP^+^ cells. (**C**) Representative TRAP staining of BMMs after 6 days of RANKL induction, indicating mature multinucleated osteoclasts. Scale bar: 50 μm. (**D**) Quantification of multinucleated TRAP^+^ cells. (**E**) Representative images of F-actin ring staining in osteoclasts on day 6 after RANKL induction. Scale bar: 50 μm. (**F**) Quantification of F-actin ring area. (**G**) Schematic illustration of the preosteoclast membrane fusion assay using dual-fluorescence labeling (PKH26/PKH67). (**H**) Representative fluorescence images of fused BMMs at day 3. Scale bar: 100 μm. (**I**) Quantification of membrane merge rate. (**J**) Relative mRNA expression of Rho family GTPases *Rac1*, *Cdc42*, and *Pak* under *Gdf15*-knockout conditions. (**K**) Relative mRNA expression of *Rac1*, *Cdc42*, and *Pak* following GDF15 overexpression. Data are presented as mean ± SD. Statistical analysis was performed using 2-tailed Student’s *t* tests. **P < 0.01, ***P < 0.001.

To elucidate the molecular mechanisms by which GDF15 regulates osteoclast differentiation, we isolated bone marrow–derived mononuclear cells (BMMs) from *Gdf15^fl/fl^* and *Gdf15* cKO mice. These BMMs were cultured with macrophage colony-stimulating factor (M-CSF) and RANKL to induce osteoclastogenesis, and TRAP staining was performed on days 3 and 6. At day 3, no substantial difference in the number of TRAP^+^ preosteoclasts was observed between the *Gdf15^fl/fl^* and *Gdf15* cKO groups, indicating that GDF15 does not affect the early commitment or proliferation of osteoclast precursors (Figure 7, A and B). However, by day 6, the number of mature multinucleated osteoclasts was reduced in the *Gdf15* cKO group, suggesting that GDF15 plays a critical role in later stages of osteoclast differentiation (Figure 7, C and D). Actin ring formation, a hallmark of osteoclast function, was assessed via fluorescence staining. *Gdf15* deficiency markedly impaired actin ring structure and reduced actin ring area in mature osteoclasts (Figure 7, E and F). To determine whether GDF15 regulates preosteoclast fusion, we performed a cell fusion assay using dual membrane dyes PKH67 (green) and PKH26 (red) to label separate preosteoclast populations. Coculture and subsequent imaging revealed that *Gdf15* knockout impaired preosteoclast fusion, as evidenced by a reduced number of fused cells (Figure 7, G–I). Consistently, RT-PCR analysis revealed lower mRNA expression of osteoclastogenesis-related genes — including *cathepsin K*, *DC-Stamp*, *Nfatc1*, and *Trap* — in *Gdf15* cKO cells, further confirming GDF15’s role in promoting osteoclast maturation (Supplemental Figure 10, A–D). These findings suggest that GDF15 facilitates osteoclastogenesis predominantly by enhancing the fusion of preosteoclasts.

**Figure 8:**
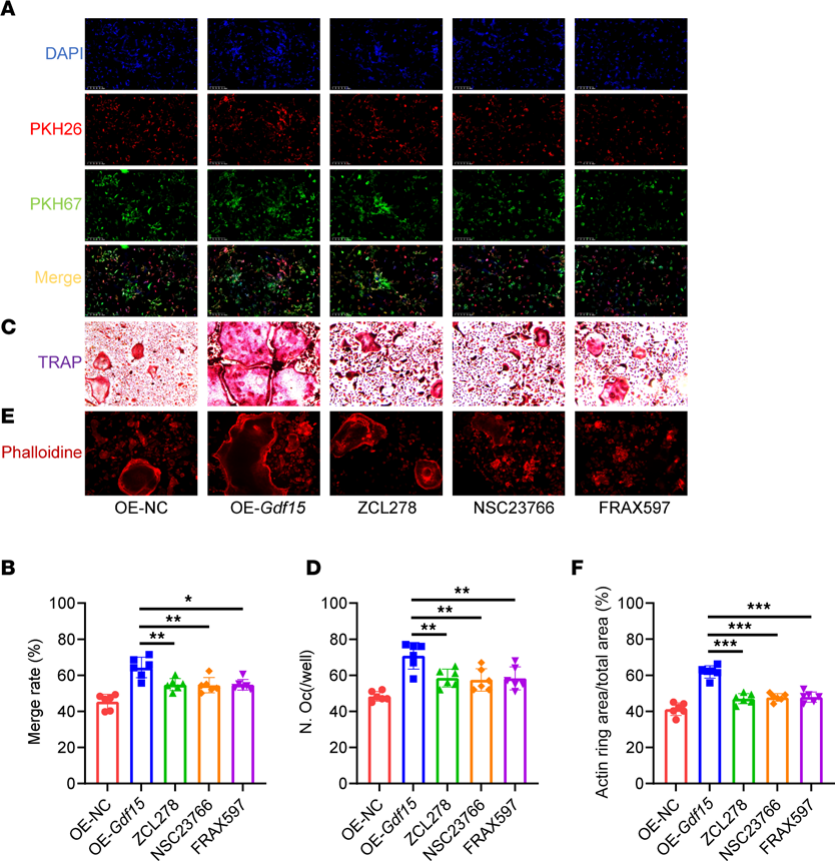
Inhibition of Rho GTPases alleviates osteoclast overactivation induced by GDF15 overexpression. (**A**) Representative dual-fluorescence images of BMMs on day 3 after GDF15 overexpression or inhibition, following RANKL induction. Scale bar: 100 μm. (**B**) Quantification of membrane merge rate following GDF15 overexpression and treatment with specific inhibitors: ZCL278 (*Cdc42* inhibitor), NSC23766 (*Rac1* inhibitor), and FRAX597 (*Pak* inhibitor). (**C**) Representative TRAP staining images of BMMs at day 6 after RANKL induction and pharmacological inhibition. Scale bar: 50 μm. (**D**) Quantification of multinucleated TRAP^+^ cells after inhibitor treatment. (**E**) Representative F-actin ring staining images under Gdf15 overexpression with or without inhibitors. Scale bar: 50 μm. (**F**) Quantification of F-actin ring area. Data are presented as mean ± SD. Statistical analysis was performed using 2-tailed ANOVA with Tukey’s test for differences among groups. *P < 0.05, **P < 0.01, ***P < 0.001.

GDF15 has been previously reported to regulate cytoskeletal dynamics via activation of small Rho GTPases, particularly *Rac1* and *Cdc42* (26), which are known to mediate osteoclast morphology and motility (27, 28). *Pak*, a downstream effector of *Rac1*/*Cdc42*, is also involved in cytoskeletal reorganization during osteoclast differentiation (29). To explore whether GDF15 acts through this signaling axis, we assessed the expression of *Rac1*, *Cdc42*, and *Pak* under RANKL stimulation. *Gdf15* knockout reduced the expression of all 3 genes (Figure 7J). Conversely, lentiviral overexpression of GDF15 in BMMs (Supplemental Figure 11, A–C) upregulated *Rac1*, *Cdc42*, and *Pak* expression under RANKL stimulation (Figure 7K). To confirm the functional relevance of these GTPases in GDF15-mediated osteoclast fusion, preosteoclasts were treated with specific inhibitors: ZCL278 (*Cdc42* inhibitor), NSC23766 (*Rac1* inhibitor), and FRAX597 (*Pak* inhibitor). While GDF15 overexpression enhanced preosteoclast fusion, treatment with these inhibitors abolished the GDF15-induced increase in fusion efficiency (Figure 8, A and B). Furthermore, TRAP staining demonstrated that the formation of mature osteoclasts induced by GDF15 overexpression was also impaired by these inhibitors (Figure 8, C and D). Actin ring staining revealed that GDF15 overexpression enhanced actin ring area, a phenotype reversed by inhibition of *Rac1*, *Cdc42*, or *Pak* (Figure 8, E and F). RT-PCR analysis confirmed that GDF15-induced upregulation of osteoclast-related genes (*cathepsin K*, *DC-Stamp*, *Nfatc1*, *Trap*) was suppressed following pharmacological inhibition of the *Rac1/Cdc42/Pak* pathway (Supplemental Figure 12, A–D). Taken together, these results demonstrate that GDF15 promotes osteoclast differentiation and maturation by enhancing preosteoclast fusion, primarily through activation of the *Rac1/Cdc42/Pak* signaling pathway.

### Targeting GDF15 could suppress the overactivation of osteoclastogenesis in the endplates and alleviated the progression of early endplate degeneration.

Based on the above findings, we hypothesized that therapeutically targeting GDF15 may offer a novel strategy for the treatment of MC-associated LBP. To investigate this, we generated a humanized GDF15 mouse model (B6-hGDF15), in which the endogenous coding region of the murine *Gdf15* gene was replaced with the corresponding human *GDF15* coding sequence via gene editing (Supplemental Figure 13A), thereby enabling tissue-specific expression of human GDF15 protein in mice. RT-qPCR analysis confirmed that human *GDF15* was robustly expressed in the liver, heart, brain, colon, and bone marrow of B6-hGDF15 mice, whereas no such expression was detected in WT controls. Conversely, expression of murine *Gdf15* was observed in these same tissues in WT mice but was absent in B6-hGDF15 mice, confirming successful replacement (Supplemental Figure 13D). Moreover, B6-hGDF15 mice displayed normal growth parameters, including body weight, length, and organ development, compared with WT controls, indicating that humanized GDF15 does not interfere with general development (Supplemental Figure 13, B, C, and E).

**Figure 9:**
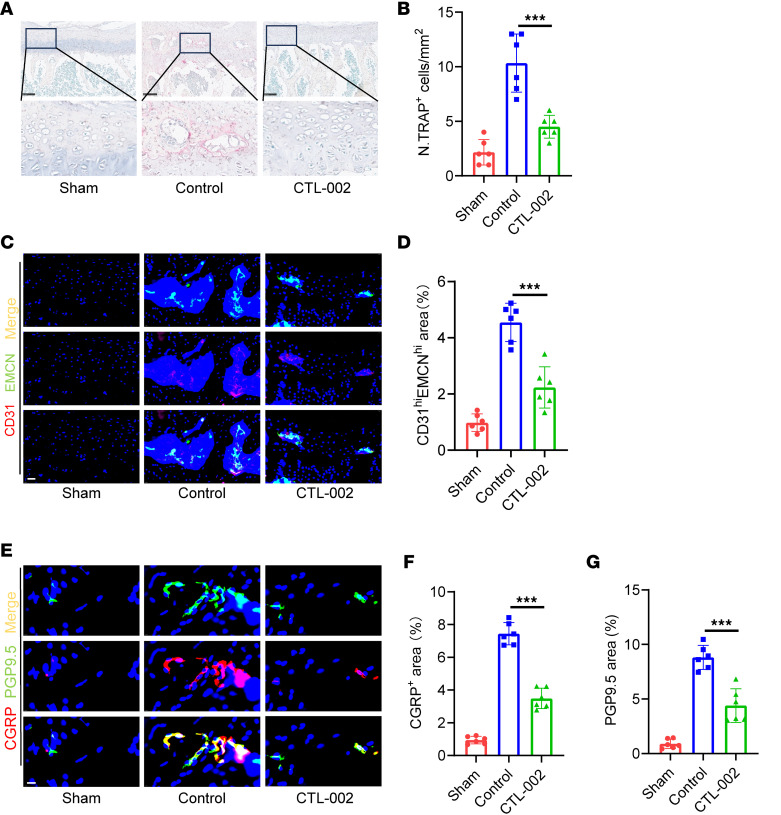
Targeting GDF15 suppresses the overactivation of osteoclastogenesis in the endplates. (**A**) Representative TRAP staining images of sham and LSI-operated mice in both control IgG or anti-GDF15 mAb treatment groups. Scale bars: 100 μm. (**B**) Quantitative analysis of the number of TRAP^+^ cells in endplates. (**C**) Representative immunofluorescence images of CD31 (red), endomucin (green), and CD31^hi^EMCN^hi^ (yellow) cells of sham and LSI-operated mice in both control IgG or anti-GDF15 mAb treatment groups. Scale bars: 20 μm. (**D**) Quantitative analysis of the areas of CD31^hi^EMCN^hi^ (yellow) cells in endplates (**E**) Representative immunofluorescence images of CGRP^+^and PGP9.5^+^ cells of sham and LSI-operated mice in both control IgG or anti-GDF15 mAb treatment groups. Scale bars: 10 μm. (**F** and **G**) Quantitative analysis of the areas of CGRP^+^ and PGP9.5^+^ cells in endplates. Data are presented as mean ± SD. Statistical analysis was performed using 2-tailed ANOVA with Tukey’s test for differences among groups. ***P < 0.001.

To assess the therapeutic potential of GDF15 inhibition, B6-hGDF15 mice were administered anti-GDF15 monoclonal antibody (CTL-002) weekly following LSI surgery. Immunohistochemical analysis revealed a marked increase in GDF15 expression within the endplates at 8 weeks after LSI, which was attenuated by CTL-002 treatment (Supplemental Figure 14, A and B). TRAP staining showed that neutralization of GDF15 reduced the number of TRAP^+^ osteoclasts in the endplate region (Figure 9, A and B), accompanied by decreased osteoclast activity markers TRACP-5b and CTX-1 (Supplemental Figure 15, A and B). Furthermore, CTL-002 treatment led to a reduction in the number of type H vessels as well as PGP9.5^+^ and CGRP^+^ sensory nerve fibers in the endplates, indicating suppression of both angiogenesis and nociceptive nerve infiltration (Figure 9, C–G). These data suggest that GDF15 neutralization mitigates osteoclast-driven pathological changes in early endplate degeneration.

**Figure 10:**
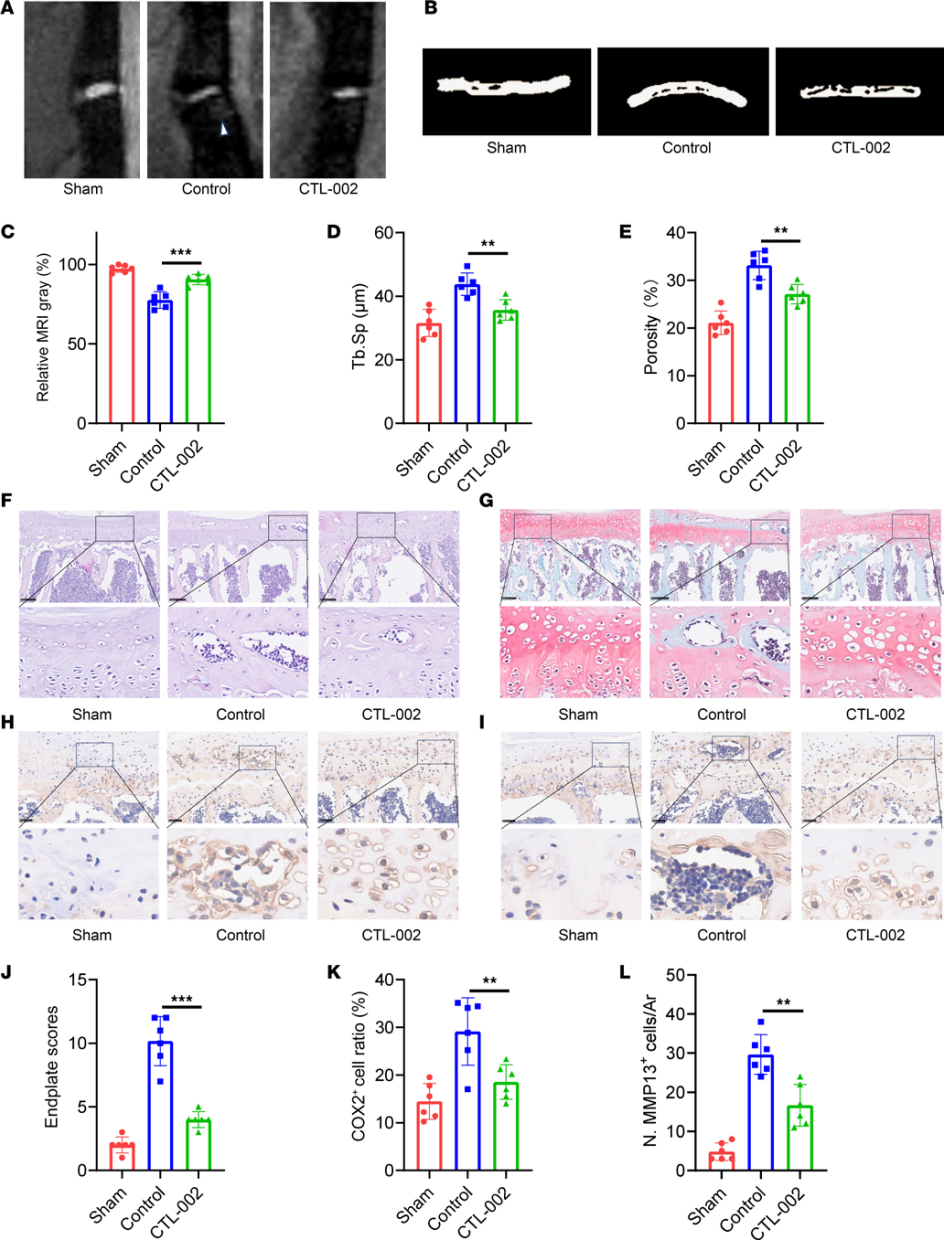
Targeting GDF15 alleviates the early endplate degeneration. (**A** and **C**) T2-weighted images of sham mice and mice after LSI surgery and after treatment with control IgG or anti-GDF15 mAb and MRI analysis. (**B**) Representative 3D micro-CT images of sham mice and mice after LSI surgery and after treatment with control IgG or anti-GDF15 mAb. (**D** and **E**) Quantitative analysis of the total porosity and trabecular separation (Tb. Sp) determined by micro-CT. (**F**) Representative images of H&E staining of sham mice and mice after LSI surgery and after treatment with control IgG or anti-GDF15 mAb. Scale bars: 100 μm. (**G**) Representative images of safranin O and fast green staining of sham mice and mice after LSI surgery and after treatment with control IgG or anti-GDF15 mAb. Scale bars: 100 μm. (**J**) Endplate scores based on safranin O and fast green staining. (**H**) Representative images of COX2 staining. Scale bars: 50 μm. (**K**) Quantitative analysis of COX2^+^ cells. (**I**) Representative images of MMP13 staining of sham mice and mice after LSI surgery and after treatment with control IgG or anti-GDF15 mAb. Scale bars: 100 μm. (**L**) Quantitative analysis of MMP13^+^ cells. Data are presented as mean ± SD. Statistical analysis was performed using 2-tailed ANOVA with Tukey’s test for differences among groups. **P < 0.01, ***P < 0.001.

Behavioral testing demonstrated that mice receiving CTL-002 exhibited improved pain-related behaviors compared with untreated LSI controls (Supplemental Figure 16, A–F). Consistent with these findings, T2-weighted MRI scans revealed reduced inflammatory signal intensity in the endplates of GDF15-neutralized mice (Figure 10, A and C). Micro-CT analysis confirmed decreased endplate destruction and reduced trabecular bone loss in the treatment group (Figure 10, B, D, and E). Histological evaluation using H&E and safranin O/fast green staining showed preservation of endplate architecture and decreased degenerative changes following GDF15 neutralization (Figure 10, F, G, and J). Immunohistochemical and RT-PCR analysis revealed markedly decreased expression of the cartilage catabolic enzymes COX2 and MMP13 (Figure 10, H, I, K, and L, and Supplemental Figure 17, A and B) in the treatment group.

In conclusion, these results demonstrate that targeting GDF15 effectively suppresses osteoclast overactivation and downstream degenerative events in the endplate microenvironment. This highlights GDF15 as a promising therapeutic target for the treatment of early endplate degeneration and MC-associated LBP.

## Discussion

CLBP specifically refers to discomfort experienced in the lower back, sacral region, and buttocks, representing the leading cause of disability and incurring substantial economic costs (30, 31). Current treatment modalities primarily focus on activity modification, surgical interventions, and pharmacological agents aimed at alleviating pain; however, these approaches do not effectively address the progression of LBP (32, 33). Most researchers have concentrated on elucidating the pathogenesis of LBP in relation to sensory innervation associated with the degeneration of lumbar intervertebral discs (34). Notably, lumbar intervertebral disc degeneration often occurs asymptomatically (35, 36). Recent studies have indicated that early stages of MCs, particularly type 1, are more prevalent in patients with CLBP than in asymptomatic individuals, suggesting a correlation between early endplate degeneration and the manifestation of back pain (37). In our study, we collected endplate tissues from patients undergoing lumbar fusion surgery who exhibited various MCs. Our findings revealed that inflammatory responses and bone resorption activities were markedly elevated in patients classified as MC-1 and MC-2, indicating a link between these types and heightened inflammation and osteoclastogenesis within the endplate. While the roles of inflammation and immune cell activity in MCs have been recently investigated, the specific contribution of osteoclast activation to these changes has yet to be reported.

Research has demonstrated that changes in vertebral biomechanics, particularly repetitive mechanical loading, can induce microfractures in the endplate and sub-endplate bone, leading to the development of fibrovascular granulation tissue (38, 39). This process is associated with increased vascular density and a heightened presence of sensory nerve fibers, both of which are critical etiological factors in MCs (40). To elucidate the pathological mechanisms driving early endplate degeneration, we conducted LSI surgery through bilateral facet joint transection, establishing a mouse model of endplate degeneration, as described previously (9, 41, 42). Imaging and histomorphological analyses revealed substantial degeneration of the endplates in mice, with observable symptoms of early endplate degeneration emerging 4–8 weeks after surgery. Furthermore, our findings indicated a notable increase in the number of osteoclasts within the endplates following LSI surgery over this 8-week period. Recent studies have highlighted that increased nerve innervation and type H angiogenesis, driven by osteoclast activity in porous endplates, are critical pathological changes contributing to endplate sclerosis, degeneration, and LBP (43–45). Subsequently, we assessed the expression of type H vessels and pain-related nerve innervation within the endplates, revealing an increase in both parameters. These results suggest that the overactivation of osteoclasts within the endplate serves as a primary factor initiating early endplate degeneration.

Several studies have indicated that osteoclasts are overactivated due to aberrant mechanical loading on the endplates (46–48). However, the specific factors contributing to the excessive activation of osteoclasts within the endplate remain largely unexplored. GDF15 was originally identified from activated macrophage cell line clones and functions as an autocrine regulatory molecule in macrophages. It belongs to the TGF-β superfamily. GDF15 primarily plays a role in organ growth, cellular repair, and joint degeneration. Under physiological conditions, GDF15 is expressed at low levels in all tissues except the placenta. In contrast, under pathological conditions, such as inflammation or traumatic stress, GDF15 expression is upregulated in the presence of TGF-β, IL-1β, TNF-α, and other stimulatory factors (49, 50). Numerous studies have recently shown that elevated levels of GDF15 are associated with the progression of various arthritic diseases. For instance, Wang et al. demonstrated that GDF15 can facilitate the progression of osteoarthritis by promoting angiogenesis (17). Additionally, Esalatmanesh et al. reported elevated serum levels of GDF15 in patients with rheumatoid arthritis (51, 52). Furthermore, GDF15 expression has been linked to bone erosion in ankylosing spondylitis (53). Nader et al. reported markedly elevated levels of GDF15 in individuals with CLBP, which aligns well with our findings of increased GDF15 expression in endplates during early degeneration and its potential role in modulating pain and osteoclastogenesis (54). These findings indirectly support our results, highlighting the pivotal role of GDF15 in the LBP. However, the mechanisms of GDF15 in LBP has yet to be elucidated. In this study, we observed that GDF15 was overexpressed in the endplates during early endplate degeneration. Notably, the knockout of GDF15 in monocytes resulted in a reduction of osteoclast numbers as well as decreased CD31^hi^Emcn^hi^ angiogenesis and pain neurogenesis in the endplates, ultimately alleviating pain symptoms and endplate degeneration. This suggests that GDF15 contributes to osteoclast activation within the endplate during the early stages of endplate degeneration.

Although the role of GDF15 in osteoclast fusion is still being elucidated, recent evidence suggests that it may modulate cytoskeletal dynamics and cell fusion processes through regulation of Rho family GTPases, including *Rac1*, *Cdc42*, and *RhoA*. These GTPases are well known for their critical roles in actin cytoskeleton reorganization, cell-cell contact formation, and intracellular signaling, which are necessary for fusion of preosteoclasts and osteoclast multinucleation (27, 29). Several studies have demonstrated that activation of *Rac1* and *Cdc42* facilitates the fusion of mononuclear osteoclast precursors, while *RhoA* activity needs to be tightly regulated to allow proper cell fusion (55, 56). GDF15 has been shown in other cell systems (e.g., tumor cells and macrophages) to influence actin remodeling and cell motility via modulation of small GTPase activity, suggesting a similar mechanism may operate in osteoclast precursors (57). In our study, we found that the upregulation of GDF15 could enhance the expression or activity of these GTPases, thus promoting the fusion of preosteoclasts and contributing to increased bone resorption activity, suggesting GDF15 may act as an upstream regulator in this process.

Nonetheless, several limitations should be acknowledged. The behavioral assays used do not specifically capture discogenic pain and may reflect general nociceptive responses. The LSI model, while valuable, may not fully represent the multifactorial nature of human Modic pathology. Moreover, the upstream regulation of GDF15 and its interaction with other inflammatory pathways remains to be explored. Future studies should refine pain models, explore upstream regulatory mechanisms of GDF15, and assess the therapeutic potential of targeting GDF15 using neutralizing antibodies or small molecule inhibitors. Multi-omics approaches may also uncover additional cell populations and signaling pathways contributing to endplate degeneration and pain.

## Methods

### Sex as a biological variable.

Specifically, for the human MC samples, sex was not considered as a biological variable during sample selection. In the animal experiments, our study exclusively examined male mice; it is unknown whether the findings are relevant for female mice.

### Patient samples.

This study involved the collection of human endplate samples from patients undergoing lumbar disc herniation surgery (*n* = 40). Clinical sample inclusion criteria included that samples were from patients who were hospitalized and underwent lumbar fusion surgery due to lumbar disc herniation or lumbar spinal stenosis, meeting surgical indications between January 2016 and December 2022. Exclusion criteria for clinical samples included a history of prior spinal surgery; presence of spinal deformities or tumors; metabolic bone diseases, such as osteoporosis; and comorbid psychiatric disorders or cognitive impairments that could affect functional assessments. The experimental group consisted of patients with LBP symptoms, a visual analogue score (VAS) ≥ 4, and lumbar MRI showing MCs in the vertebrae above or/and below the fusion segment. The experimental group consisted of patients with LBP symptoms, a visual analogue score (VAS) ≥ 4, and lumbar MRI showing MCs in the vertebrae above or/and below the fusion segment.

The study population included individuals with or without confirmed MCs as identified by spine MRI. According to the criteria and classifications described by de Roos and Modic, Udby et al. suggested that when different or mixed types of MC are present within the same functional spinal unit, the MC with the most clinical significance should be described first (i.e., MC-1, followed by MC-2, and then MC-3) (58–60). Therefore, our diagnosis of mixed-type MC in certain clinical patients is based on the most clinically significant MC. During specimen collection, MRI was used preoperatively to locate the MC-affected tissue to ensure more accurate sampling of the corresponding MC-affected tissue. Intraoperatively, spinal canal decompression was first performed, followed by the complete removal of disc tissue and exposure of the upper and lower endplates. The Medtronic O-arm navigation (StealthStation S8) system was used for pedicle screw placement and to simultaneously identify the sampling site. Endplate tissue was scraped from the designated location using a curette for RT-PCR analysis.

### RNA isolation and quantitative RT-PCR.

Total RNA was isolated from the various groups using TRIzol reagent (Invitrogen), and residual genomic DNA was removed by DNase I treatment (Thermo Fisher Scientific) according to the manufacturer’s protocol. RNA concentration and purity were determined using a spectrophotometer (NanoDrop 2000, Thermo Fisher Scientific). Reverse transcription was performed using PrimeScript RT MasterMix (TaKaRa Bio) to synthesize complementary DNA from 1 μg total RNA.

Quantitative real-time PCR (RT-qPCR) was conducted using SYBR Green qPCR Master Mix (Applied Biosystems) on a StepOnePlus Real-Time PCR System (Applied Biosystems). Primers were designed to span exon-exon junctions to avoid genomic DNA amplification. The RT-qPCR protocol included an initial denaturation at 95°C for 5 minutes, followed by 40 cycles of amplification (95°C for 15 seconds and 60°C for 60 seconds). Melting curve analysis was performed at the end of each run (95°C for 15 seconds and 60°C for 60 seconds) to confirm the specificity of the amplification products.

All reactions were run in triplicate, and expression levels were normalized to the internal control gene β-actin using the 2^–ΔΔCt^ method. No-template controls and no–reverse transcription (no-RT) controls, in which reverse transcriptase was omitted during cDNA synthesis, were included to exclude contamination and amplification of genomic DNA. The primer sequences are shown in Table 1.

### Mouse models and in vivo experiments.

The *Gdf15*-floxed mouse line (catalog NM-CKO-232914) used in our experiments was purchased from Shanghai Model Organisms, and B6-hGDF15 mice were provided by Cyagen Biotechnology Company. The transgenic mice used in this study were bred on a C57BL/6 pure strain background and kept under specific pathogen–free conditions. They were housed in a controlled environment with a temperature of approximately 22°C and a 12-hour light/dark cycle. WT littermates of the same sex were used as controls for all experiments. Eight-week-old C57BL/6 male mice were used for the in vivo experiments. In general, mice were first anesthetized by pentobarbital sodium (10 mg/kg, intraperitoneally). Then, animals from both the LSI and sham models were operated on, with excision of the L3–L5 spinous process to result in the LSI as reported previously (41). For the sham group, the operations were conducted by only detachment of the posterior paravertebral muscles from L3–L5 vertebral body. Therapeutic administration of control IgG (AstraZeneca, NIP228, 10 mg/kg) or anti-GDF15 mAb (10 mg/kg, MEC, HY-P99100) was administrated by tail vein injection 3 times a week for 2 weeks.

### Micro-CT analyses.

For quantitative micro-CT assays, mice were anesthetized with overdose of pentobarbital sodium. The lumbar spines were dissected and stored in 100% ethyl alcohol for 24 hours and then stored in PBS solutions. All of the data were analyzed by a high-resolution micro-CT (Skyscan 1076, Bruker). As reported previously (61), the scanner was set at a resolution of 8 m per pixel, a voltage of 80 kV, and a current of 124 A. The 3D models were analyzed by CTAn and CTVol. Two hundred section planes below the growth plate were observed.

### Immunofluorescence and histomorphometry.

For immunofluorescence images, the full lumbar spines were removed from the mice and fixed in 4% paraformaldehyde at 4°C for 72 hours. Then, the spine tissues were decalcified with EDTA (0.5 M; pH 7.4) solutions for 72 hours with continuous shaking. After that, the tissues were washed for at least 3 times with PBS solutions and embedded in paraffin or OCT compound. Sections of 30 μm thickness were used for CD31 and EMCN immunofluorescence images. All of the experiments were conducted following the procedures of previous studies (61, 62). Rat anti-EMCN antibody (1:100; sc-65495, Santa Cruz Biotechnology) and rabbit anti-CD31 antibody (1:20; ab28364, Abcam) were used for CD31 and EMCN immunofluorescence images. Specifically, we used a negative control in which the primary antibody was replaced with a species- and isotype-matched nonimmune IgG at the same concentration. These controls were processed under identical conditions as the experimental samples. No specific fluorescence signal was detected in the negative control samples, confirming the specificity of the primary antibody staining. Sections of 5 μm thickness were used for safranin O, fast green, and TRAP (Sigma-Aldrich) staining. We calculated endplate scores as described previously (63, 64). Sections of 5 μm thickness were used for the CGRP sensory nerve immunofluorescence staining (1:100, ab81887, Abcam). All of the experiments were conducted following the methods of previous studies (65).

### ELISA.

The serum levels of CTX-1 and TRACP-5 were examined by ELISA using a CTX-1 ELISA kit (NBP2- 69074, Novus) and a TRACP-5b ELISA kit (EK7661, SAB). All ELISAs were performed in accordance with the manufacturer’s instructions.

### RNA sequencing of monocytes from endplates and subchondral bone of endplate.

We first obtained the endplates and subchondral bone tissue using tissue scissors. The samples were then prepared as a cell suspension in a solution containing sterile PBS (pH 7.2) and 2 mM EDTA, kept at 2°C–8°C. The cell suspension was subsequently filtered through a 100 μm mesh to remove bone debris and cell clumps. Following this, we performed gradient centrifugation, first centrifuging the cell suspension at 500*g* for 20 minutes at 25°C. The supernatant was then removed, and the pellet was centrifuged at 300*g* for 10 minutes at 20°C. CD14 microbeads were added to the cell suspension and mixed thoroughly, followed by incubation at 4°C for 15 minutes. After adding the buffer, the suspension was centrifuged at 300*g* for 10 minutes. The cell pellet was resuspended and sorted using a flow cytometer. The cells were then treated with Trizol reagent to extract total RNA, which was sequenced using the BGI sequencing platform. Differentially expressed genes were identified using the criteria of FDR < 0.05 and log_2_FC > 1 or log_2_FC < –1. To elucidate the biological significance of these differentially expressed genes, GO analysis was performed using the R-package cluster profiler. Additionally, pathway analysis was conducted on these genes to determine the substantially affected pathways, using the KEGG database via the R-package clusterProfiler. Visualization of the data, including a circular heatmap of selected differentially expressed genes, a classification summary plot of KEGG pathway analysis, and a GOChord plot, was created using an online platform for data analysis and visualization (http://www.bioinformatics.com.cn). The data have been submitted to Sequence Read Archive (SRA) and are fully accessible to the public. The accession number for the dataset is PRJNA1328439.

### Cell culture and transfection.

Bone marrow stromal cells were isolated from the femurs of 8-week-old C57BL/6 mice. The cells were then cultured for 3 days in α-MEM medium supplemented with 15% fetal bovine serum and 30 ng/mL M-CSF. Adherent cells were subsequently collected and seeded into 24-well plates. The cells were incubated at 37°C in a humidified atmosphere with 5% CO_2_. Transfection was performed by circ^–^ control, circ-GDF15. The forward oligonucleotide sequence is TAATACGACTCACTATAGGGCCACCATGCCCGGGCAAGAACTCAG, and the reverse oligonucleotide is TCTGAGATGAGTTTTTGTTCTATGCAGTGGCAGTCTTTGGCT.

### Osteoclast differentiation experiments and actin ring staining.

The bone marrow stromal cells were flushed out from the femurs of 8-week-old C57BL/6 mice as described previously (66). Then, cells were cultured for 3 days in α-MEM medium with 15% fetal bovine serum and 30 ng/mL M-CSF. Adherent cells were then collected and seeded into a 24-well plates. Cells were then incubated with M-CSF (30 ng/mL) and RANKL (100 ng/mL) for 3 (preosteoclast) and 6 (osteoclast) days, respectively. Then, TRAP staining (Sigma-Aldrich) was conducted following the manufacturer’s instructions. TRAP^+^ cells with more than 3 nuclei were regarded as mature osteoclasts. For actin-ring formation assay, the osteoclasts were fixed and stained with Phalloidin (Alexa Fluor 488 Phalloidin, catalog 8878, Thermo Scientific; dilution 1:60) at room temperature for 1 hour to visualize actin rings. The number of actin rings was analyzed by ImageJ (NIH) software.

### Osteoclastic fusion assays.

The preosteoclasts were obtained as described above. The preosteoclasts were labeled with the red fluorescent dye PKH26 (Sigma-Aldrich) or the green florescent dye PKH67 (Sigma-Aldrich) following the manufacturer’s instructions. Then, the 2 groups of preosteoclasts were seeded together on the 24-well plate for 2 hours. The liquid supernatant was removed, and fluorescence microscopy was conducted. The membrane merge rate was evaluated using the ImageJ as reported previously (67).

### Behavioral testing.

All behavioral tests were conducted by the same investigator, who was blinded to the research groups.

Pressure tolerance was detected by the vocalization thresholds (as a nociceptive threshold) using a force gauge as described previously (65). C57BL/6 mice were gently restrained and received the pressure force by a sensor on their skin above the L4–L5 spine. A cumulative increase of pressure force (50 g/s) was conducted on the mice until the animals showed an audible vocalization.

Spontaneous running activity was measured by several indicators (including distance traveled, mean speed, and maximum speed) by the activity wheels. C57BL/6 mice were placed in cages that were similar to their home cages, and the wheels of the device could be used by the mice from all directions. The software of the equipment could show the real-time information of the spontaneous activity of the mice.

The pain hypersensitivity in response to external stimulation was detected by hind paw withdrawal frequency (PWF) using the von Frey test with 0.7 mN and 3.9 mN. C57BL/6 mice were put in a transparent plastic cage, which was put on a metal mesh grid. The midplantar position of the mice’s hind paw was stimulated by 0.7 mN or 3.9 mN. The frequency of mechanical stimulus was 10 times at a 1-second interval. When the hind paw was withdrawn after the stimulation by von Frey filaments, the data were taken down.

### Statistics.

We performed data analyses with Graphpad Prism software. Data were presented as mean ± SD. We used unpaired 2-sample (2-tailed) *t* test to compare the data from 2 groups. We performed the 1-way ANOVA to compare the data from multiple groups. *P* < 0.05 was regarded as statistically significance for all experiments.

### Study approval.

The animal studies in this research were approved by the Ethics Committee of Shanghai Changhai Hospital. The Ethics Committee of Shanghai General Hospital approved this research (2023AWS094, Shanghai, China), and written informed consent was obtained from all patients or their relatives prior to surgery.

### Data availability.

The data have been submitted to SRA and are fully accessible to the public (accession PRJNA1328439). Values for all data points in graphs are reported in the Supporting Data Values file. Additional datasets generated and/or analyzed during the current study are available from the corresponding author upon reasonable request.

## Author contributions

JM, XL, and XZ designed this study and analyzed the data. YL and XL conducted the majority of the experiments and completed the manuscript. JW, QK, and XL participated in the experimental design and the manuscript writing. MH and ZW collected animal model samples. HJ participated in editing the manuscript. All authors approved the final version of the manuscript.

## Funding support

- Natural Science Foundation of China (82201748, 82571824, 82571824).
- Shanghai Sailing program (21YF1459200).

## Supplementary Material

Supplemental data

Unedited blot and gel images

Supporting data values

## Figures and Tables

**Table 1:**
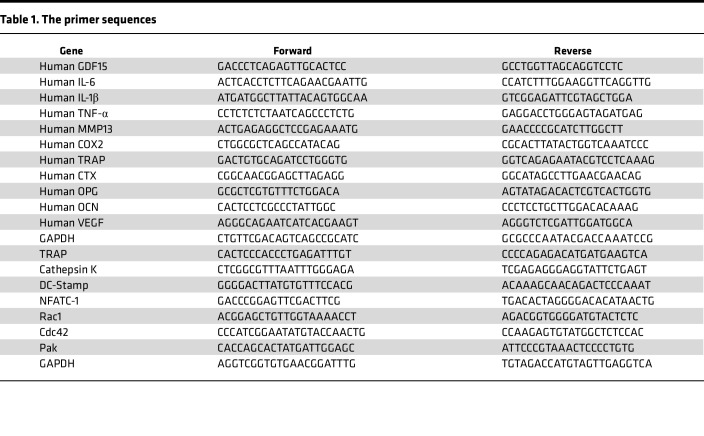
The primer sequences

## Version 1 11/11/2025

In-Press Preview

## Version 2 01/09/2026

Electronic publication

## References

[1] Choudhry NK (2022). Effect of a biopsychosocial intervention or postural therapy on disability and health care spending among patients with acute and subacute spine pain: The SPINE CARE randomized clinical trial. JAMA.

[2] Zhou Z (2022). Potential mechanisms underlying the accelerated cognitive decline in people with chronic low back pain: A scoping review. Ageing Res Rev.

[3] Wall J (2022). Incidence, prevalence and risk factors for low back pain in adolescent athletes: a systematic review and meta-analysis. Br J Sports Med.

[4] Roseen EJ (2023). Racial and ethnic disparities in the incidence of high-impact chronic pain among primary care patients with acute low back pain: a cohort study. Pain Med.

[5] Peng B (2009). Diagnosis and surgical treatment of back pain originating from endplate. Eur Spine J.

[6] Kawabata S (2024). Intradiscal administration of autologous platelet-rich plasma in patients with Modic type 1 associated low back pain: A prospective pilot study. JOR Spine.

[7] Duan Y (2024). The relationship between Modic changes and endplate sclerosis in patients with lumbar degenerative disease: a systematic review and network meta-analysis. World Neurosurg.

[8] Xu J (2024). Targeting type H vessels in bone-related diseases. J Cell Mol Med.

[9] Pan D (2024). Senescence of endplate osteoclasts induces sensory innervation and spinal pain. Elife.

[10] Degmetich S (2016). Neural innervation patterns in the sacral vertebral body. Eur Spine J.

[11] Lin CR (2006). Prostaglandin E2 receptor EP4 contributes to inflammatory pain hypersensitivity. J Pharmacol Exp Ther.

[12] Morales-Soto W (2023). Enteric glia promote visceral hypersensitivity during inflammation through intercellular signaling with gut nociceptors. Sci Signal.

[13] Liu S (2021). TRPV1 channel activated by the PGE2/EP4 pathway mediates spinal hypersensitivity in a mouse model of vertebral endplate degeneration. Oxid Med Cell Longev.

[14] Latif R (2022). Vertebral endplate changes correlate with presence of cartilaginous endplate in the herniated disc tissue: factor predicting failure of conservative treatment. Asian Spine J.

[15] Mattia L (2023). Effect of age and gender on serum growth differentiation factor 15 and its relationship to bone density and bone turnover. Bone Rep.

[16] Zhang Y (2022). Evaluating the impact of metformin targets on the risk of osteoarthritis: a mendelian randomization study. Osteoarthritis Cartilage.

[17] Weng PW (2022). Role of GDF15/MAPK14 axis in chondrocyte senescence as a novel senomorphic agent in osteoarthritis. Int J Mol Sci.

[18] He YW, He CS (2022). Association of growth and differentiation factor 15 in rheumatoid arthritis. J Inflamm Res.

[19] Suriben R (2022). Antibody-mediated inhibition of GDF15-GFRAL activity reverses cancer cachexia in mice. Nat Med.

[20] Coll AP (2020). GDF15 mediates the effects of metformin on body weight and energy balance. Nature.

[21] Herpich C (2022). The effect of dextrose or protein ingestion on circulating growth differentiation factor 15 and appetite in older compared to younger women. Nutrients.

[22] Hinoi E (2012). Positive regulation of osteoclastic differentiation by growth differentiation factor 15 upregulated in osteocytic cells under hypoxia. J Bone Miner Res.

[23] Segerer SE (2012). MIC-1 (a multifunctional modulator of dendritic cell phenotype and function) is produced by decidual stromal cells and trophoblasts. Hum Reprod.

[24] Li S (2020). GDF15 induced by compressive force contributes to osteoclast differentiation in human periodontal ligament cells. Exp Cell Res.

[25] Xue P (2024). Proton-activated chloride channel increases endplate porosity and pain in a mouse spine degeneration model. J Clin Invest.

[26] Kempf T (2011). GDF-15 is an inhibitor of leukocyte integrin activation required for survival after myocardial infarction in mice. Nat Med.

[27] Gao L (2020). The regulatory role of Rho GTPases and their substrates in osteoclastogenesis. Curr Drug Targets.

[28] Razzouk S (1999). Rac-GTPase, osteoclast cytoskeleton and bone resorption. Eur J Cell Biol.

[29] Gu J (2020). Rho-GEF trio regulates osteoclast differentiation and function by Rac1/Cdc42. Exp Cell Res.

[30] Cashin AG (2021). Efficacy, acceptability, and safety of muscle relaxants for adults with non-specific low back pain: systematic review and meta-analysis. BMJ.

[31] Ferreira GE (2021). Efficacy and safety of antidepressants for the treatment of back pain and osteoarthritis: systematic review and meta-analysis. BMJ.

[32] Bråten L (2019). Efficacy of antibiotic treatment in patients with chronic low back pain and Modic changes (the AIM study): double blind, randomised, placebo controlled, multicentre trial. BMJ.

[33] Luckhaupt SE (2019). Prevalence, recognition of work-relatedness, and effect on work of low back pain among U.S. Workers. Ann Intern Med.

[34] Foster NE (2018). Prevention and treatment of low back pain: evidence, challenges, and promising directions. Lancet.

[35] Rubinstein SM (2019). Benefits and harms of spinal manipulative therapy for the treatment of chronic low back pain: systematic review and meta-analysis of randomised controlled trials. BMJ.

[36] Vlaeyen JWS (2018). Low back pain. Nat Rev Dis Primers.

[37] Mylenbusch H (2023). Efficacy of stepped care treatment for chronic discogenic low back pain patients with Modic I and II changes. Interv Pain Med.

[38] Yu Q (2024). Finite element analysis of biomechanical investigation on diverse internal fixation techniques in oblique lumbar interbody fusion. BMC Musculoskelet Disord.

[39] Xie S (2025). Contact between leaked cement and adjacent vertebral endplate induces a greater risk of adjacent vertebral fracture with vertebral bone cement augmentation biomechanically. Spine J.

[40] Ji Z (2024). Mrgprb2-mediated mast cell activation exacerbates Modic changes by regulating immune niches. Exp Mol Med.

[41] Li H (2024). Lumbar instability remodels cartilage endplate to induce intervertebral disc degeneration by recruiting osteoclasts via Hippo-CCL3 signaling. Bone Res.

[42] Liu S (2021). A mouse model of lumbar spine instability. J Vis Exp.

[43] Tao S (2022). Red light-mediated photoredox catalysis triggers nitric oxide release for treatment of *Cutibacterium acne* induced intervertebral disc degeneration. ACS Nano.

[44] Watt NB (2017). Safety observations with 3 years of denosumab exposure: comparison between subjects who received denosumab during the randomized FREEDOM trial and subjects who crossed over to denosumab during the FREEDOM extension. J Bone Miner Res.

[45] Xu L (2016). The anti-NGF antibody muMab 911 both prevents and reverses pain behaviour and subchondral osteoclast numbers in a rat model of osteoarthritis pain. Osteoarthritis Cartilage.

[46] Wang J (2020). Osteal tissue macrophages are involved in endplate osteosclerosis through the OSM-STAT3/YAP1 signaling axis in Modic changes. J Immunol.

[47] Yaun FL (2014). Molecular actions of ovarian cancer G protein-coupled receptor 1 caused by extracellular acidification in bone. Int J Mol Sci.

[48] Perilli E (2015). Modic (endplate) changes in the lumbar spine: bone micro-architecture and remodelling. Eur Spine J.

[49] Maschalidi S (2022). Targeting SLC7A11 improves efferocytosis by dendritic cells and wound healing in diabetes. Nature.

[50] Conte M (2022). GDF15, an emerging key player in human aging. Ageing Res Rev.

[51] Esalatmanesh K (2020). The association between serum levels of growth differentiation factor-15 and rheumatoid arthritis activity. Int J Clin Pract.

[52] Song Y (2018). Increased serum levels of MIC1/GDF15 correlated with bone erosion in spondyloarthritis: a pilot study. Medicine (Baltimore).

[53] Okcu M (2022). Epicardial adipose tissue thickness and growth differentiation factor 15 in axial spondyloarthritis: A cross-sectional study. Saudi Med J.

[54] Tarabeih N (2019). Growth and differentiation factor 15 is a biomarker for low back pain-associated disability. Cytokine.

[55] Yang Y (2025). Mechanism of ITGB2 in Osteoclast Differentiation in Osteoarthritis. Cell Prolif.

[56] Zhang J (2024). Phillygenin prevents osteoclast differentiation and bone loss by targeting RhoA. Phytother Res.

[57] Zhao J (2024). Tumor-derived GDF15 induces tumor associated fibroblast transformation from BMSCs and fibroblasts in oral squamous cell carcinoma. J Cell Physiol.

[58] Udby PM (2022). A definition and clinical grading of Modic changes. J Orthop Res.

[59] de Roos A (1987). MR imaging of marrow changes adjacent to end plates in degenerative lumbar disk disease. AJR Am J Roentgenol.

[60] Modic MT (1988). Degenerative disk disease: assessment of changes in vertebral body marrow with MR imaging. Radiology.

[61] Li X (2020). Targeting actin-bundling protein L-plastin as an anabolic therapy for bone loss. Sci Adv.

[62] Xie H (2014). PDGF-BB secreted by preosteoclasts induces angiogenesis during coupling with osteogenesis. Nat Med.

[63] Boos N (2002). Classification of age-related changes in lumbar intervertebral discs: 2002 Volvo Award in basic science. Spine (Phila Pa 1976).

[64] Masuda K (2005). A novel rabbit model of mild, reproducible disc degeneration by an anulus needle puncture: correlation between the degree of disc injury and radiological and histological appearances of disc degeneration. Spine (Phila Pa 1976).

[65] Ni S (2019). Sensory innervation in porous endplates by Netrin-1 from osteoclasts mediates PGE2-induced spinal hypersensitivity in mice. Nat Commun.

[66] Song H (2019). Reversal of osteoporotic activity by endothelial cell-secreted bone targeting and biocompatible exosomes. Nano Lett.

[67] Dou C (2017). Graphene-based microRNA transfection blocks preosteoclast fusion to increase bone formation and vascularization. Adv Sci (Weinh).

