# Supplementary material for: GDF15 induces excessive activation of osteoclasts within the vertebral endplates leading to early endplate degeneration
Source: JCI Insight. 2025 Nov 11;11(1):e190598. doi: 10.1172/jci.insight.190598 (PMC12890488; doi:10.1172/jci.insight.190598)
Supplement: Supplemental data [file jciinsight-11-190598-s173.pdf]

Supporting information：

GDF15 induces excessive activation of osteoclasts within the vertebral endplates leading to early endplate degeneration

Xiaoqun Li[3*#], Jinhui Wu[2*], Qingjie Kong[1*], Miao Hu[2], Yuhong Li[3], Ziheng Wei[1], Heng Jiang[2], Xuhui Zhou[1,2#], Jun Ma[1#]

1. Department of Orthopedics, Shanghai General Hospital, Shanghai Jiao Tong University School of Medicine, Shanghai, P.R.China
2. Department of Orthopedics, Shanghai Changzheng Hospital, Naval Medical University, Shanghai, P.R.China
3. Department of Orthopedics, Shanghai Changhai Hospital, Naval Medical University, Shanghai, P.R.China

*Xiaoqun Li, Jinhui Wu, Qingjie Kong and Miao Hu contributed equally to this work.

#Corresponding author and to whom requests for offprints should be sent: Jun Ma (ma1997juns@163.com), Xuhui Zhou (xhzhouspine@163.com) and Xiaoqun Li (drlixiaoqun@163.com)

Address: Department of Orthopedics, Shanghai General Hospital, Shanghai Jiao Tong University School of Medicine, No.85 Wujin Road, Shanghai, P.R.China

Tel.:+86 21 63240090-6322

Fax:+86 21 63240825

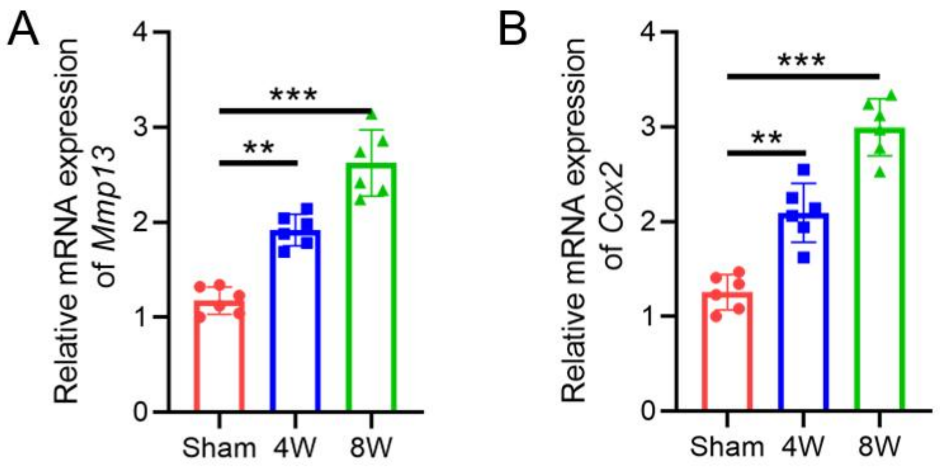

Supplementary figure 1. LSI aggravated the degeneration of endplate. (A, B) Relative mRNA expression of *Mmp13* and *Cox2* in endplates. (Data are presented as means ± SD, Statistical analysis was performed using two-tailed ANOVA with Tukey test for differences among groups. $^{*}P<0.05$, $^{**}P<0.01$, $^{***}P<0.001$).

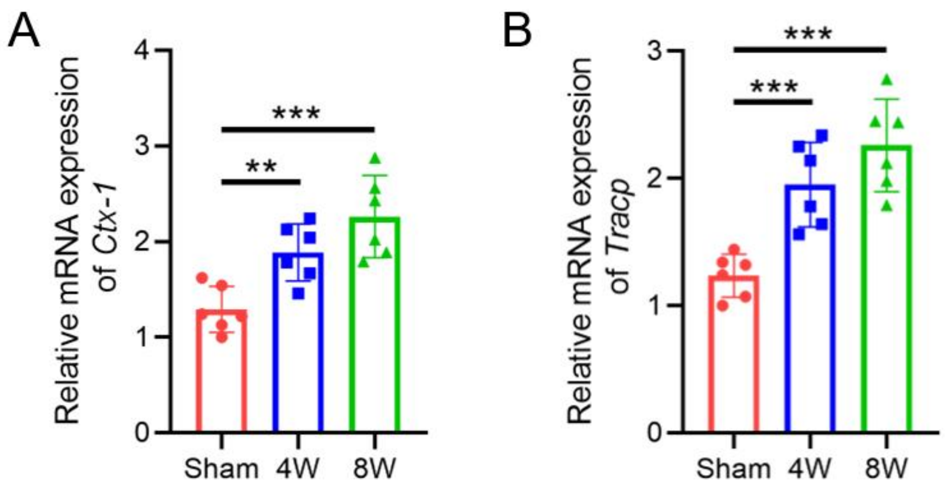

Supplementary figure 2. The osteoclastogenesis in endplates were over-activated after LSI. (A, B) Relative mRNA expression of *Ctx-1* and *Tracp* in the endplate. (Data are presented as means ± SD, Statistical analysis was performed using two-tailed ANOVA with Tukey test for differences among groups. *P<0.05, **P<0.01, ***P<0.001).

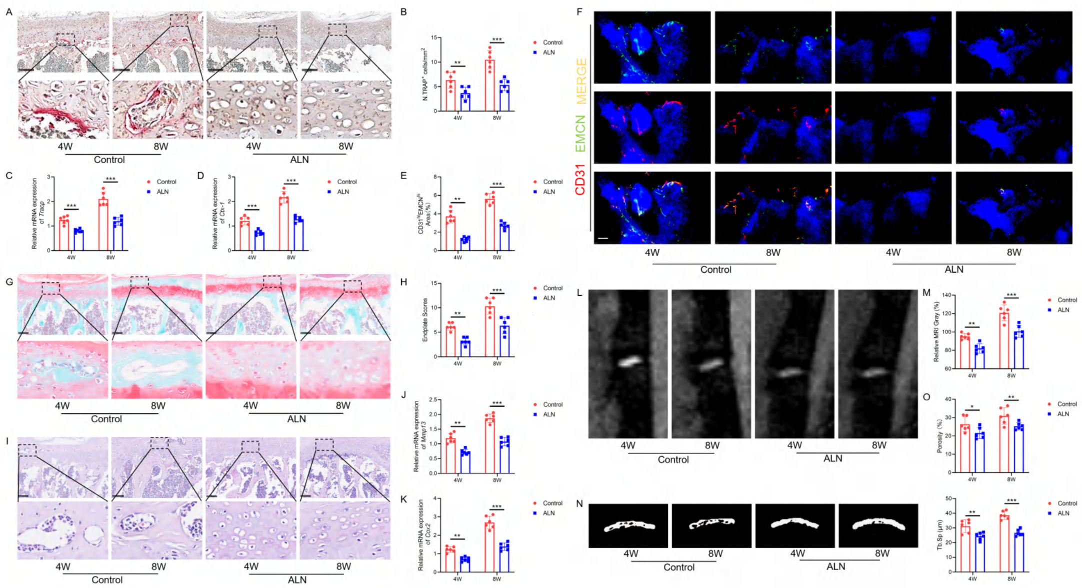

Supplementary Figure 3. Inhibition of osteoclastogenesis alleviated the endplate degeneration after LSI surgery. (A) Representative TRAP staining images. Scale bars, 100μm. **(B)** Quantitative analysis of the number of TRAP^{+} cells in endplates. **(C, D)** Relative mRNA expression of CTX-1 and TRACP. **(F)** Representative immunofluorescent images of CD31 (red), endomucin (green) and CD31^{hi}EMCN^{hi} (yellow) cells. Scale bars, 20μm. **(E)** Quantitative analysis of the areas of CD31^{hi}EMCN^{hi} (yellow) cells in endplates. **(G)** Representative images of safranin O and fast green staining of *Gdf15*$^{flox/flox}$ and *Gdf15* cKO mice after LSI or sham surgery. Scale bars, 100μm. **(H)** Endplate scores based on safranin O and fast green staining. **(I)** Representative images of Hematoxylin and eosin (H&E) staining. Scale bars, 100 μm. **(J, K)** Relative mRNA expression of *Mmp13* and *Cox2* in endplates. **(L, M)** T2-weighted images of control or ALN treatment groups, and MRI analysis. **(N)** Representative three-dimensional micro-CT images of the mice. **(O)** Quantitative analysis of the total porosity and trabecular separation (Tb. Sp) determined by micro-CT. (Data are presented as means ± SD, Statistical analysis was performed using two-tailed Student's t tests. $^{*}P<0.05$, $^{**}P<0.01$, $^{***}P<0.001$).

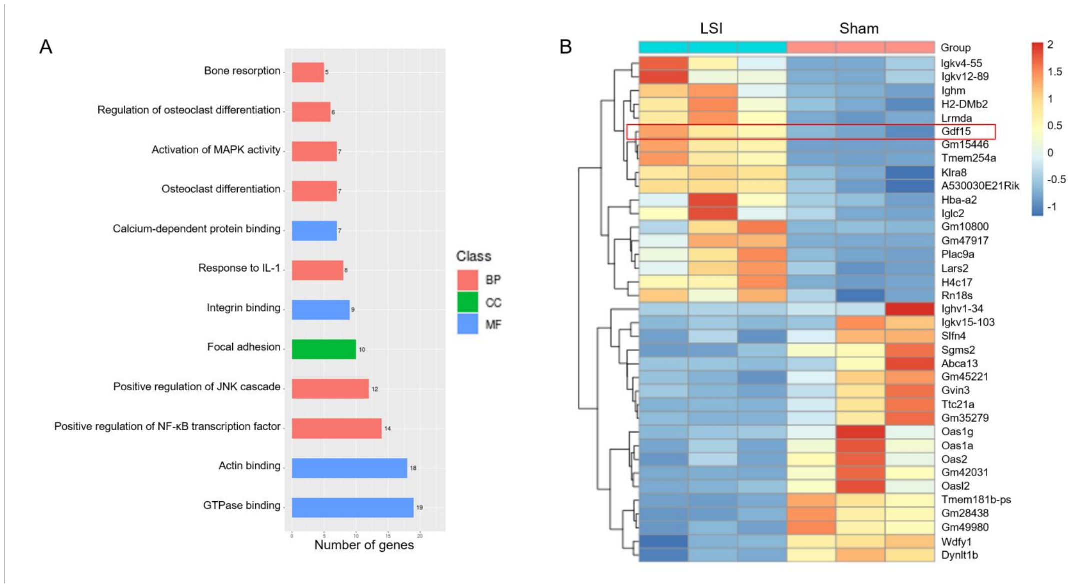

Supplementary Figure 4. RNA sequencing of monocytes isolated from the endplate and subchondral bone of both LSI-treated and sham-operated mice. (A) Kyoto Encyclopedia of Genes and Genomes (KEGG) pathway analysis of enriched signaling pathways. **(B)** Heatmap displaying altered genes in LSI vs. Sham groups.

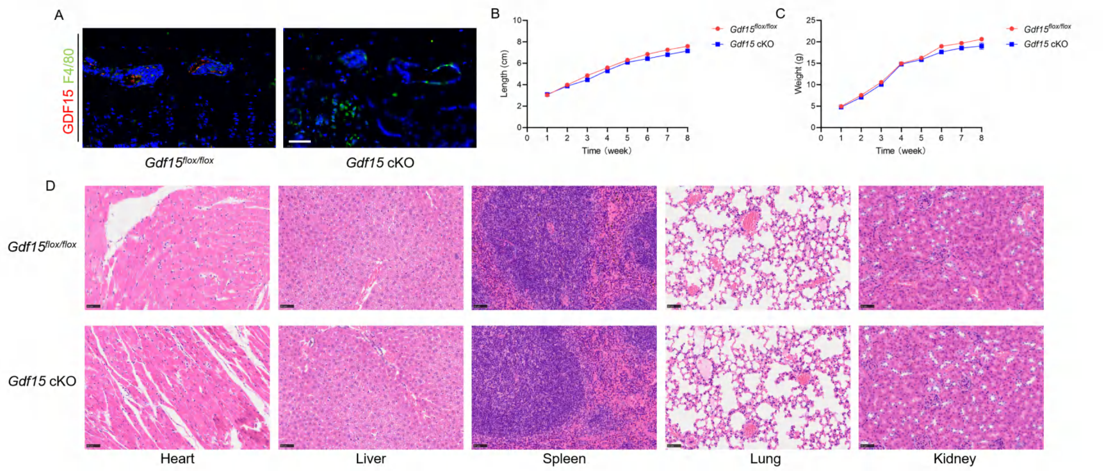

Supplementary figure 5. Construction of *Gdf15* conditional knock-out mice. (A) Representative immunofluorescent images of GDF15 (red), F4/80 (green) positive cells. Scale bars, 20μm. **(B)** Body weight of *Gdf15*$^{flox/flox}$ and *Gdf15* cKO mice within 8 weeks after birth. **(C)** Body length of *Gdf15*$^{flox/flox}$ and *Gdf15* cKO mice within 8 weeks after birth. **(D)** H&E staining of organ development at 8 weeks after birth. n = 6 per group.

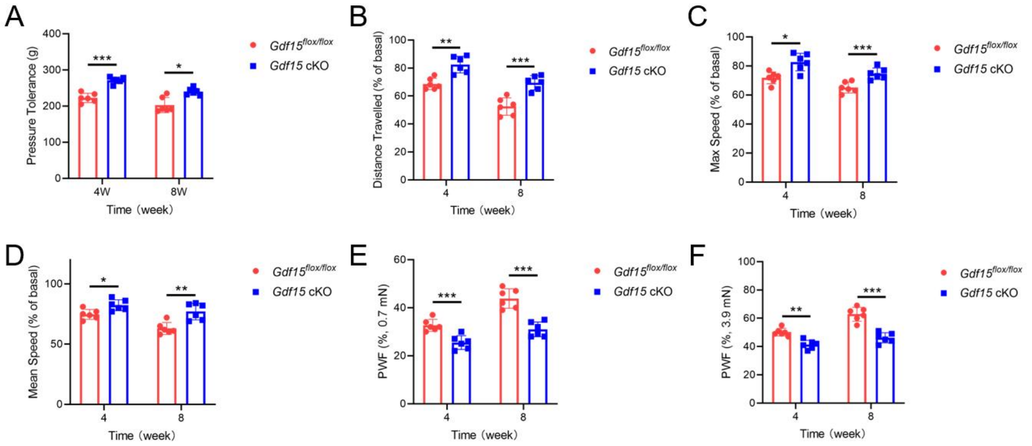

Supplementary Figure 6. The behavior experiments of *Gdf15$^{flox/flox}$* and *Gdf15* cKO mice. The hyperalgesia of the lumbar spine at different time point after LSI surgery. The Spontaneous activity at different time points was evaluated by **(A)** Pressure Tolerance. **(B)** Distance traveled. **(C)** Max speed. **(D)** Mean speed. **(E, F)** The hind paw withdrawal frequency responding to mechanical stimulation (von Frey, 0.7 mN and 3.9 mN). (Data are presented as means ± SD, Statistical analysis was performed using two-tailed Student's t tests. *P<0.05, **P<0.01, ***P<0.001).

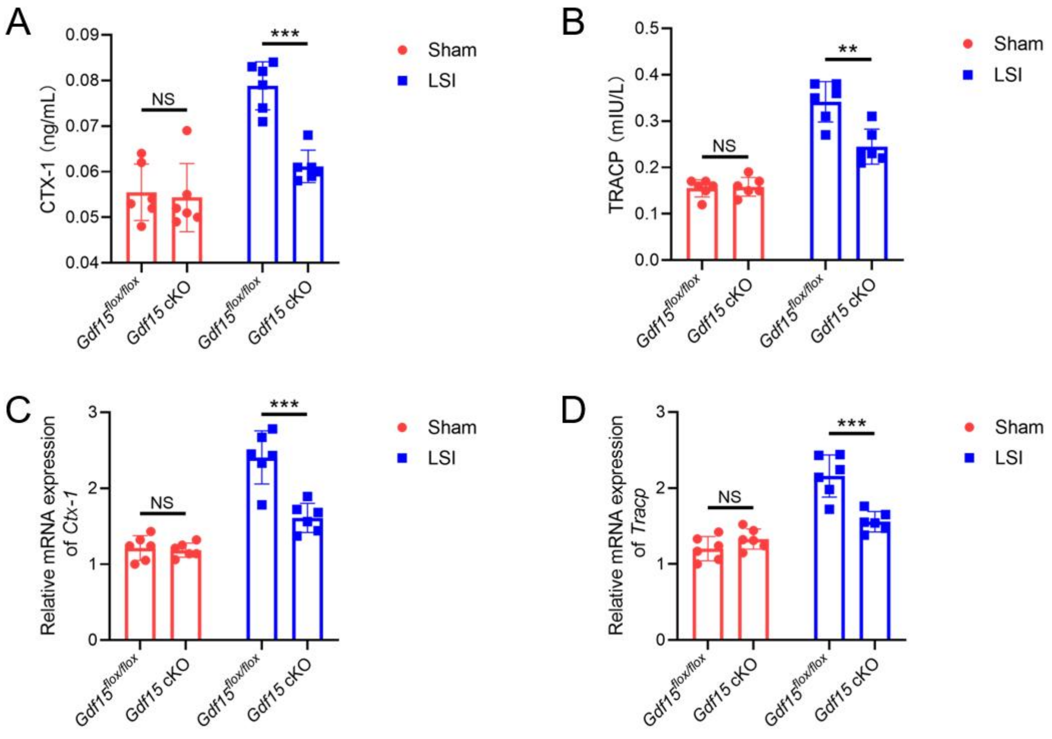

Supplementary Figure 7. Conditional knock out of *Gdf15* inhibited osteoclastogenesis

(A, B) The serum levels of CTX-1 and TRACP. **(C, D)** Relative mRNA expression of *Ctx-1* and *Tracp*. (Data are presented as means ± SD, Statistical analysis was performed using two-tailed Student's t tests. $^{*}P<0.05$, $^{**}P<0.01$, $^{***}P<0.001$, NS: not significant).

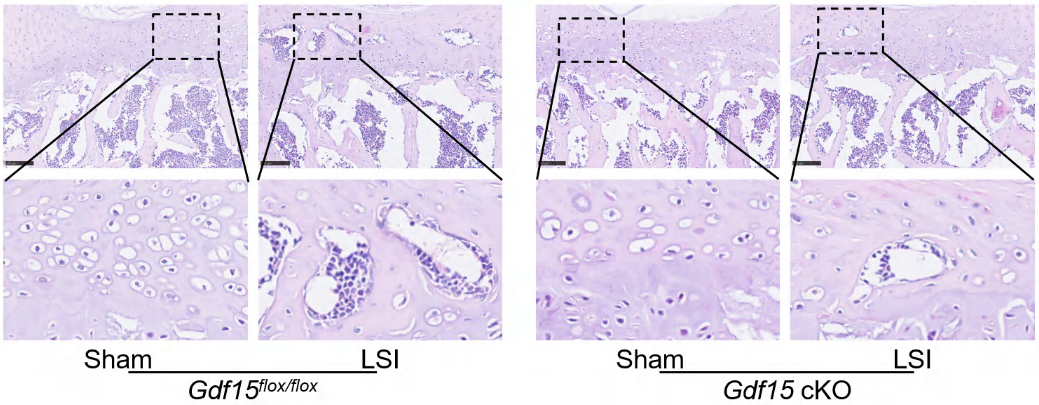

Supplementary Figure 8. Hematoxylin and eosin (H&E) staining indicated that conditional knockout of *Gdf15* alleviate the endplate damage in early endplate degeneration.

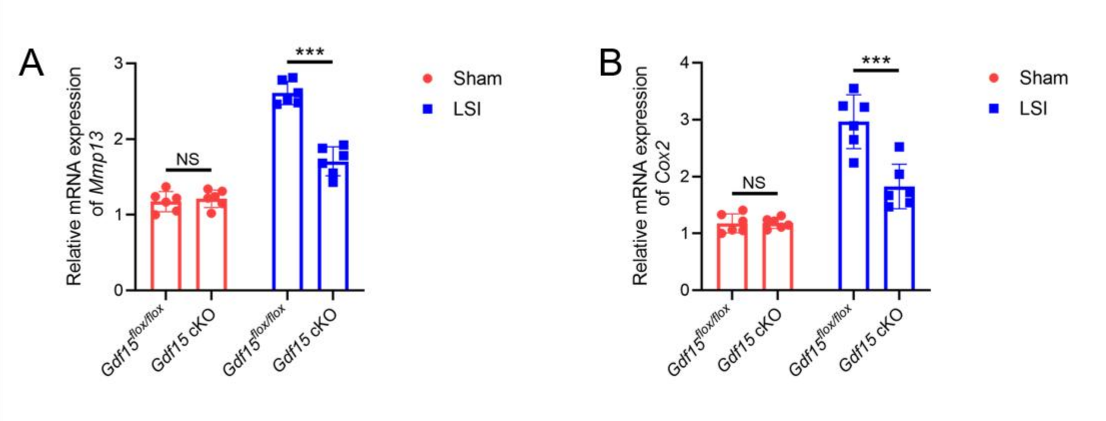

Supplementary Figure 9. Conditional knock out of *Gdf15* inhibited endplate degeneration. (A, B) Relative mRNA expression of *Mmp13* and *Cox2* in endplates. (Data are presented as means ± SD, Statistical analysis was performed using two-tailed Student's t tests. *P<0.05, **P<0.01, ***P<0.001, NS: not significant).

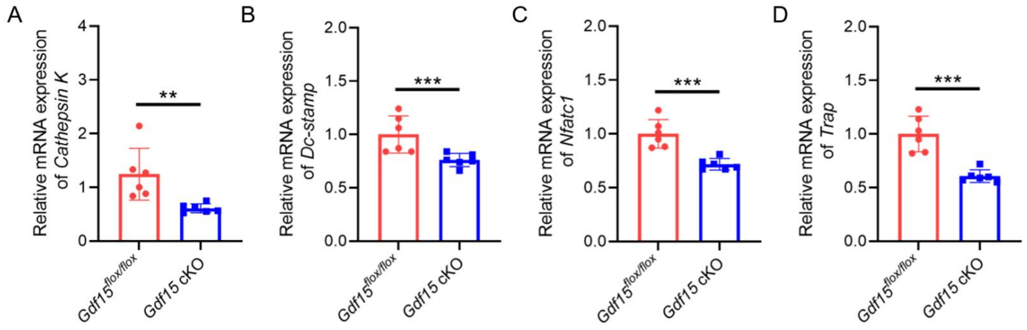

Supplementary Figure 10. ***Gdf15*** **knockout impaired the osteoclastogenesis. (A-D)** Relative mRNA expression of osteoclastogenesis-related genes: *Cathepsin K, Dc-stamp, Nfatc1,* and *Trap*. (Data are presented as means ± SD, Statistical analysis was performed using two-tailed Student's t tests. *P<0.05, **P<0.01, ***P<0.001).

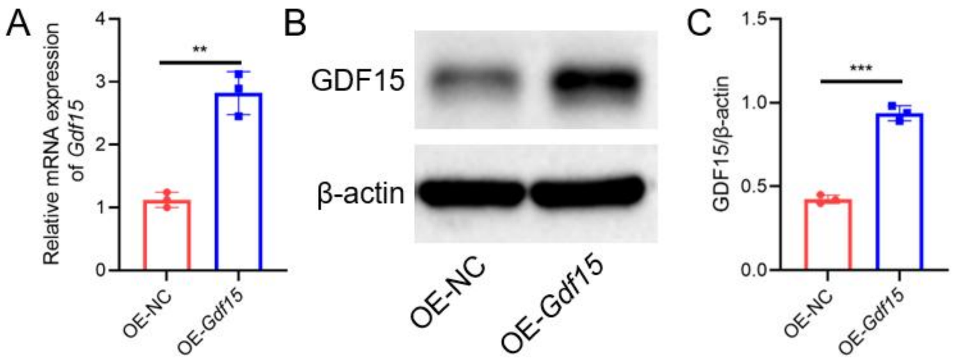

Supplementary Figure 11. Overexpression of GDF15 in BMMs. (A) Gene expression of *Gdf15*. **(B, C)** Western blot analysis of GDF15. (Data are presented as means ± SD, Statistical analysis was performed using two-tailed Student's t tests. *P<0.05, **P<0.01, ***P<0.001).

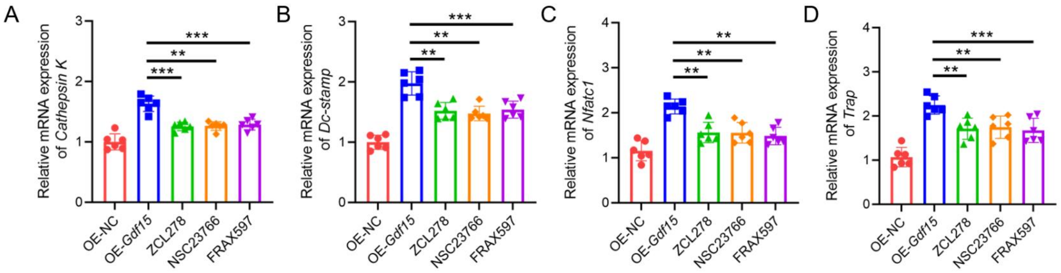

Supplementary Figure 12. GDF15-induced upregulation of osteoclast-related genes was suppressed following pharmacological inhibition of the *Rac1/Cdc42/Pak* pathway
(A-D) Relative mRNA expression of osteoclastogenesis-related genes: *Cathepsin K, Dc-stamp, Nfatc1,* and *Trap*. (Data are presented as means ± SD, Statistical analysis was performed using two-tailed ANOVA with Tukey test for differences among groups. *P<0.05, **P<0.01, ***P<0.001).

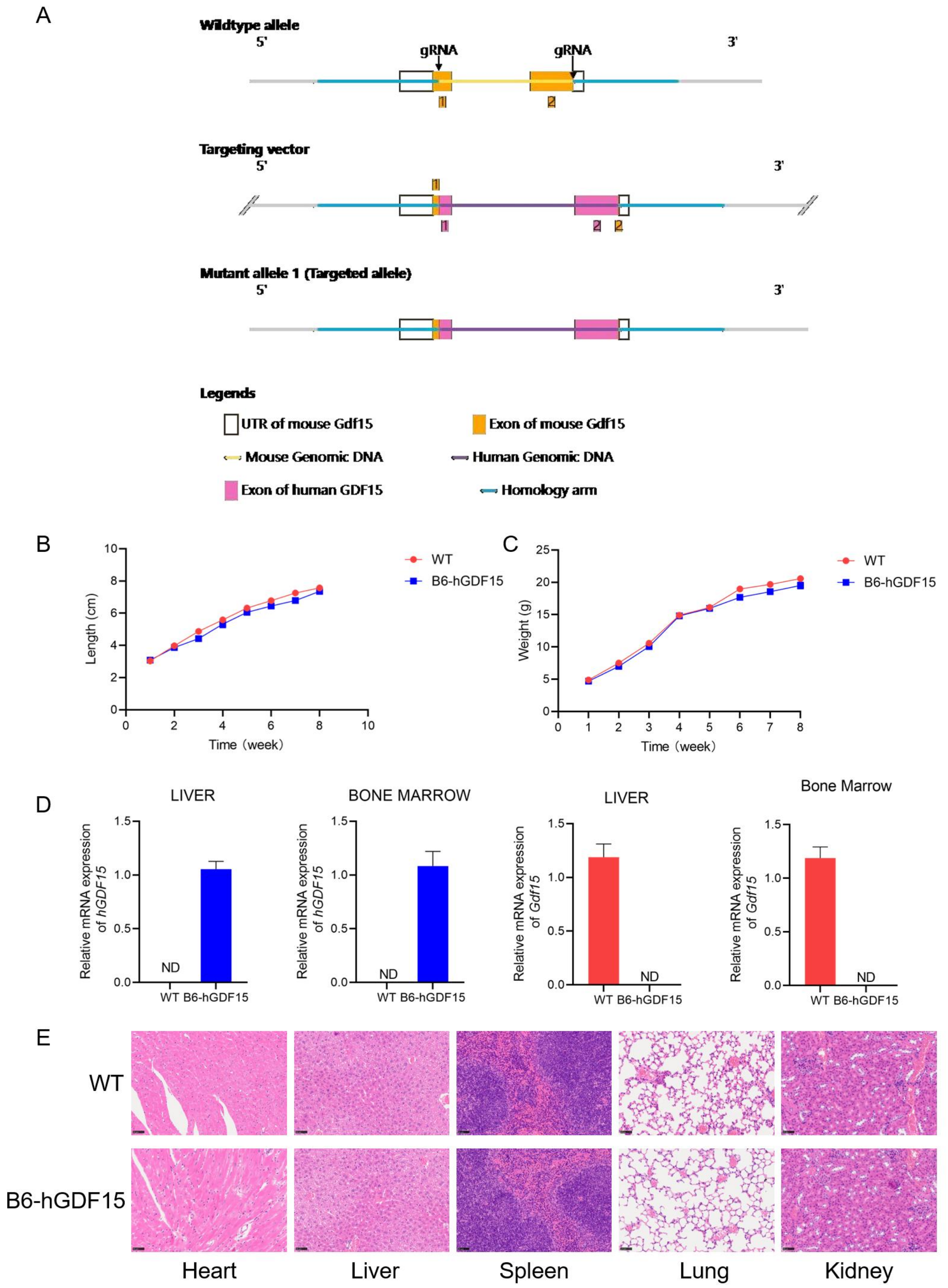

Supplementary figure 13. Construction of B6-hGDF15 mice. (A) The schematic diagram of design strategy of B6-hGDF15 mice. (B) Body weight of WT and B6-hGDF15 mice within 8 weeks after birth. (C) Body length of WT and B6-hGDF15 mice within 8 weeks after birth. (D) RT-PCR of hGDF15, Gdf15 in liver and bone marrow. (E) H&E staining of organ development at 8 weeks after birth. (Data are presented as means ± SD, Statistical analysis was performed using two-tailed Student's t tests. $^{*}P<0.05$, $^{**}P<0.01$, $^{***}P<0.001$).

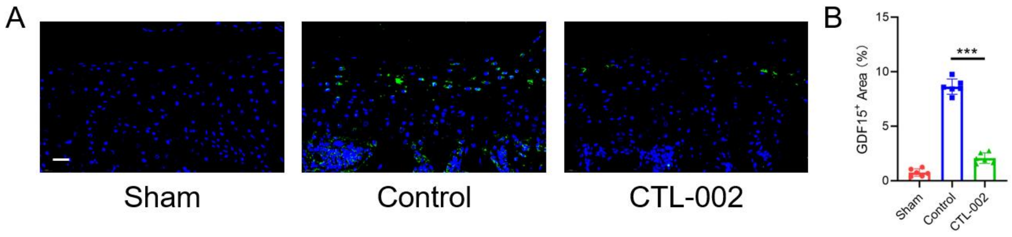

Supplementary figure 14. The expression of GDF15 in endplates of B6-hGDF15 mice. **(A)** Representative immunofluorescent images of GDF15 positive cells. Scale bars, 20μm. **(B)** Quantitative analysis of the GDF15 positive cells in endplates. (Data are presented as means ± SD, Statistical analysis was performed using two-tailed ANOVA with Tukey test for differences among groups. $^{*}P<0.05$, $^{**}P<0.01$, $^{***}P<0.001$).

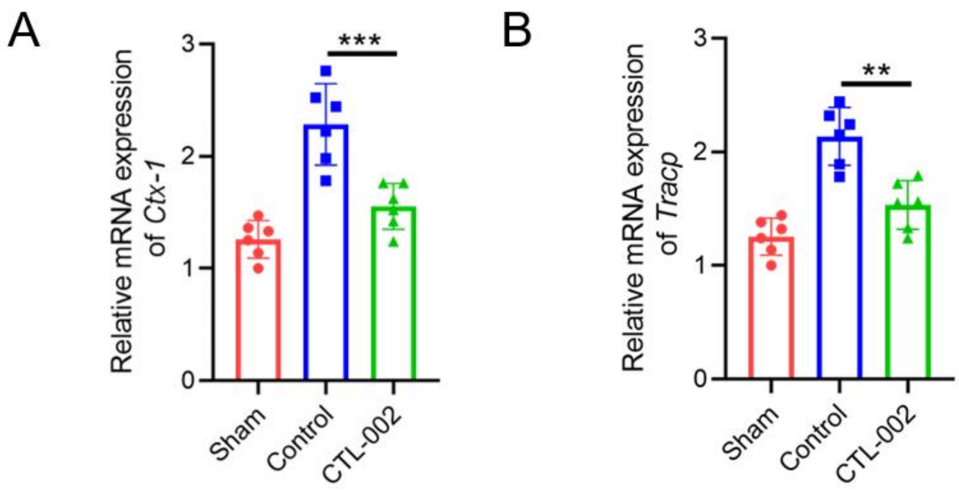

Supplementary figure 15. Targeting GDF15 could suppress the over-activation of osteoclastogenesis in the endplates. (A, B) Relative mRNA expression of *Ctx-1* and *Tracp*. (Data are presented as means ± SD, Statistical analysis was performed using two-tailed ANOVA with Tukey test for differences among groups. *P<0.05, **P<0.01, ***P<0.001).

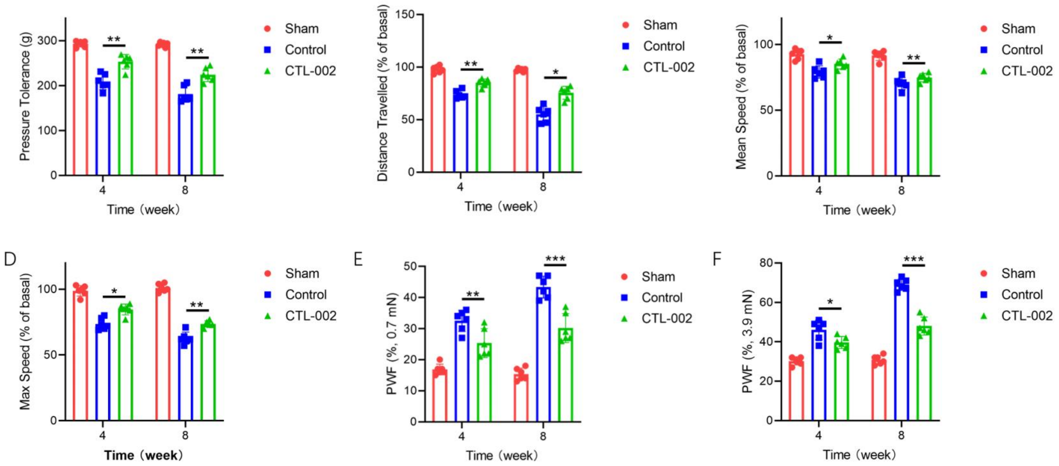

Supplementary Figure 16. The behavior experiments of WT and B6-hGDF15 mice. The hyperalgesia of the lumbar spine at different time points after LSI surgery. The Spontaneous activity at different time points was evaluated by **(A)** Pressure Tolerance. **(B)** Distance traveled. **(C)** Max speed. **(F)** Mean speed. **(E, F)** The hind paw withdrawal frequency responding to mechanical stimulation (von Frey, 0.7 mN and 3.9 mN). n = 6 per group. (Data are presented as means ± SD, Statistical analysis was performed using two-tailed ANOVA with Tukey test for differences among groups. *P<0.05, **P<0.01, ***P<0.001).

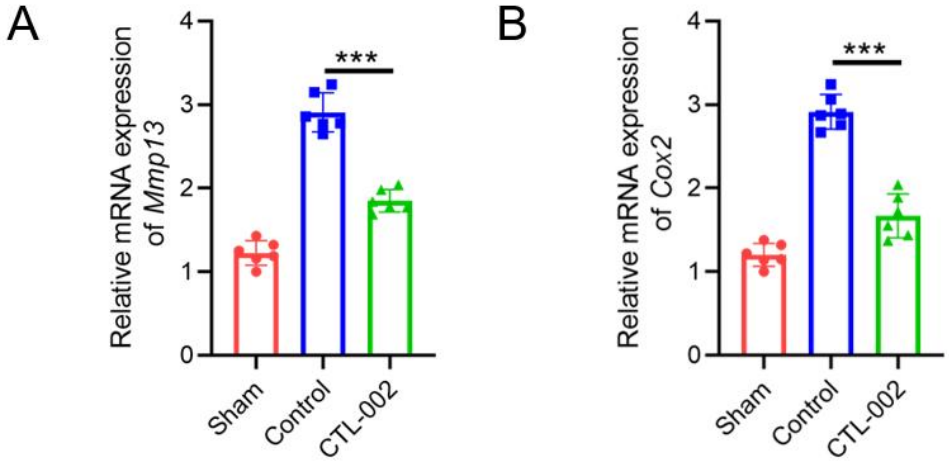

Supplementary figure 17. Targeting GDF15 alleviated the early endplate degeneration. (A, B) Relative mRNA expression of *Mmp13* and *Cox2*. (Data are presented as means ± SD, Statistical analysis was performed using two-tailed ANOVA with Tukey test for differences among groups. *P<0.05, **P<0.01, ***P<0.001).
