# Supplementary figures and images for: GDF15 induces excessive activation of osteoclasts within the vertebral endplates leading to early endplate degeneration
Source: JCI Insight. 2025 Nov 11;11(1):e190598. doi: 10.1172/jci.insight.190598 (PMC12890488; doi:10.1172/jci.insight.190598)

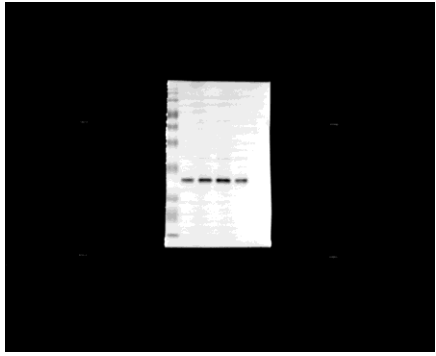

VEGF

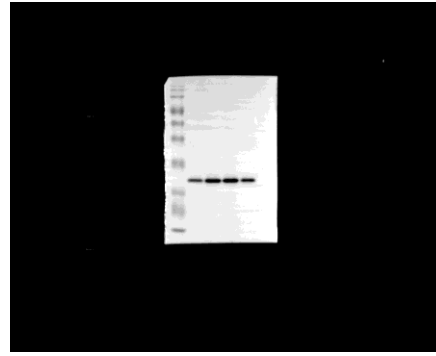

TNF- $\alpha$

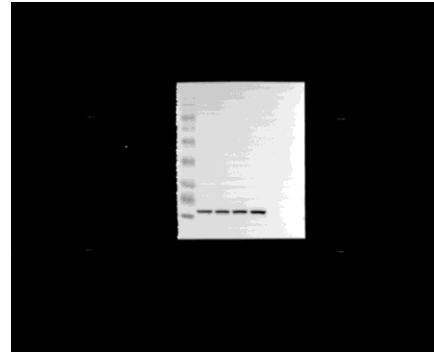

OCN

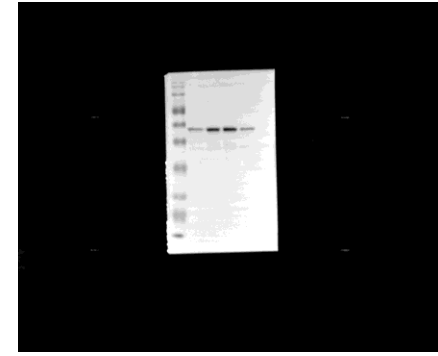

MMP13

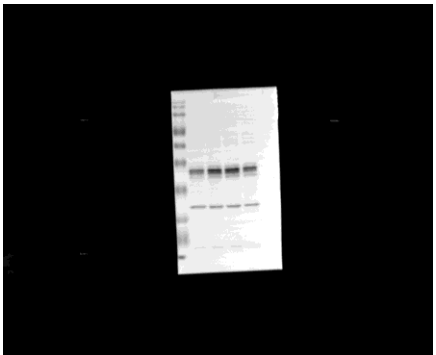

CTSK

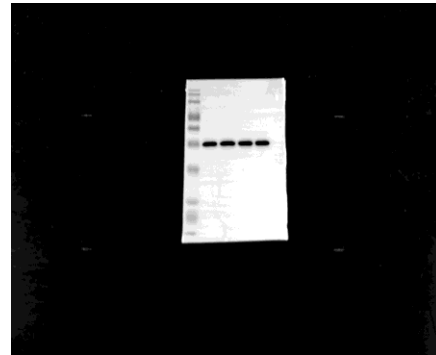

Actin

Fig 1L

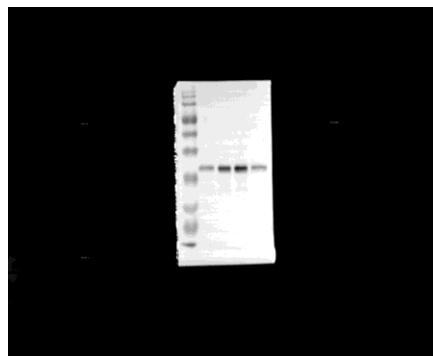

GDF15

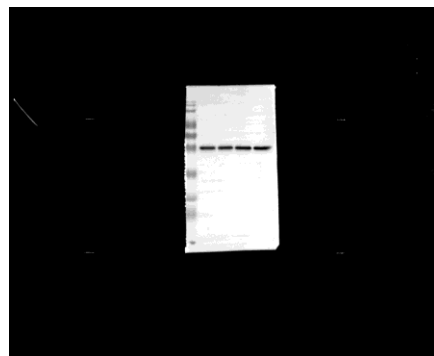

ACTIN

Fig 3R

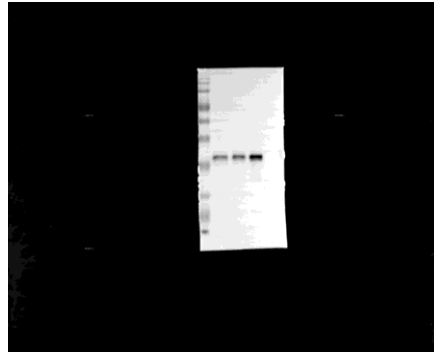

GDF15

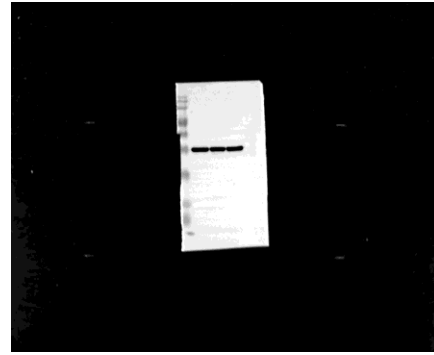

ACTIN

Sup fig 9 B

Supplement: Unedited blot and gel images [file jciinsight-11-190598-s174.pdf]
